# Growth and Persistence of an Aerobic Microbial Community in Wyoming Bentonite MX-80 Despite Anoxic *in situ* Conditions

**DOI:** 10.3389/fmicb.2022.858324

**Published:** 2022-04-25

**Authors:** Niels Burzan, Roberta Murad Lima, Manon Frutschi, Andrew Janowczyk, Bharti Reddy, Andrew Rance, Nikitas Diomidis, Rizlan Bernier-Latmani

**Affiliations:** ^1^Environmental Microbiology Laboratory, École Polytechnique Fédérale de Lausanne, Lausanne, Switzerland; ^2^Bioinformatics Core Facility, Swiss Institute of Bioinformatics, Lausanne, Switzerland; ^3^Jacobs Engineering Group Inc., Critical Missions Solutions, Harwell Science and Innovation Campus, Didcot, United Kingdom; ^4^National Cooperative for the Disposal of Radioactive Waste, Wettingen, Switzerland

**Keywords:** bentonite, radioactive waste, canister, microbiome, anoxic, *in-situ*, sulfate-reducing bacteria, microbial induced corrosion

## Abstract

Microbial activity has the potential to enhance the corrosion of high-level radioactive waste disposal canisters, which, in the proposed Swiss deep geological repository, will be embedded in bentonite and placed in the Opalinus Clay (OPA) rock formation. A total of 12 stainless steel cylindrical vessels (referred to as modules) containing bentonite were deployed in an anoxic borehole in OPA for up to 5.5 years. Carbon steel coupons were embedded in the bentonite. Individual modules were retrieved after 1, 1.5, 2.5, and 5.5 years. Enumeration of aerobic and anaerobic heterotrophs and sulfate-reducing bacteria (SRB) revealed microbial growth for 1.5 years followed by a decline or stagnation in microbial viability. It was surprising to observe the growth of aerobic heterotrophs followed by their persistent viability in bentonite, despite the nominally anoxic conditions. In contrast, SRB numbers remained at very low levels. DNA-based amplicon sequencing confirmed the persistence of aerobes and the relatively low contribution of anaerobes to the bentonite microbiome. Bentonite dry density, *in situ* exposure time, and bioavailable trapped oxygen are observed to shape the bentonite microbial community in the clay.

## Introduction

The safe storage of radioactive waste produced by nuclear power plants is an environmental challenge faced by societies utilizing electricity from nuclear fission. Deep geological repositories are envisioned by many countries as the most promising path to safe and long-term disposal (Ewing et al., 2016). The discovery of the natural nuclear reactor Oklo and its subsequent characterization confirms the suitability of geological disposal for nuclear waste (Gauthier-Lafaye, 1986, 2002). In Switzerland, Nagra (National Cooperative for the Disposal of Radioactive Waste) is considering the Opalinus Clay (OPA) rock formation as the host rock for the construction of deep geological repositories. Carbon steel is the current reference material for the canisters containing spent fuel and high-level radioactive waste, and the current disposal concept envisions a gallery backfilled with Wyoming bentonite with a dry density of 1.45 g/cm^3^ around the canisters. In this concept (Nagra, 2009), the cylindrical canister will rest on a base of compacted bentonite blocks with a dry density of 1.55 g/cm^3^.

This study builds on a long-term, *in situ* experiments designed to evaluate the corrosion of carbon steel embedded in Wyoming bentonite and deployed in OPA. An initial time point of 1.5 years that included microbial viability investigations has previously been communicated (Smart et al., 2017). That study provided initial insights into the *in situ* corrosion of carbon steel in bentonite and the viability of microorganisms in three different bentonite dry densities. However, it lacked microbial DNA profiling after 1.5 years of deployment in OPA. Here, we report on three additional time-points (1, 2.5, and 5.5 years) and the DNA-based characterization of the microbial community. Bentonite buffers, due to their significant swelling potential when in contact with water, are expected to create conditions unsuitable for microbial growth, such as low water content, limited pore space, and nutrient availability restricted by diffusion processes (Pedersen et al., 2000; Stroes-Gascoyne, 2010; Rättö and Itävaara, 2012). Three bentonite dry densities were employed in this study. The goal was to provide *in situ* corrosion rates and insights regarding the viability and persistence of microbes in bentonite. Of particular interest are sulfate-reducing bacteria (SRB), whose activity has been shown to lead to microbiologically influenced corrosion (MIC) of carbon steel (Černoušek et al., 2020). It is expected that a considerable number of bacterial cells will remain viable despite the harsh conditions expected post-repository closure, which include increased pressure, heat, and irradiation (Haynes et al., 2018). Recently, an *in situ* study (Engel et al., 2019b) featuring an identical design to the present one, but deployed within a granite host rock environment for ∼1 year, reported a persistent microbial community organized along a spatial gradient between the surrounding anoxic rock porewater and the bentonite. The borehole porewater community was dominated by SRB belonging to *Desulfosporosinus* and *Desulfovibrio*. The surface of the module was colonized primarily by *Pseudomonas stutzeri*, while the bentonite itself included genera of aerobic bacteria, *Streptomyces* sp. and *Xanthomonas* sp. The interpretation was that these organisms grew slowly and could be remnant (extracellular) DNA adsorbed to clay particles in bentonite. The authors suggested that further studies should include cultivation and activity assays to confirm the viability of microbes within deployed bentonite cylinders.

The goal of this study was to determine whether microorganisms grew in the bentonite under long-term, near-repository conditions and to investigate whether they had the potential to enhance the rate of *in situ* carbon steel corrosion. In addition, the origin of microorganisms living in bentonite, whether from the host rock and porewater or from the bentonite itself, was also considered. An understanding of which microorganisms grow or persist in bentonite is essential to determining the potential for MIC. Here, employing a combination of classical cultivation techniques and DNA-based tools, confidence in the composition of the bentonite microbiome could be established. The findings point to a microbiome that includes a large fraction of aerobic heterotrophic microorganisms and that exhibits a closer relationship to the bentonite microbiome before deployment than to that of the surrounding anoxic borehole. Thus, we demonstrate the presence of a viable aerobic community in bentonite deployed in an anoxic environment, showing growth or persistence over the tested timeframes and bentonite dry densities examined.

## Materials and Methods

### Experimental Design

Experimental modules consisted of stainless steel cylinders (25 cm tall, outer diameter of 12.6 cm), lined with a sintered, porous stainless steel filter, and filled with Wyoming bentonite. The bentonite was prepared at one of three dry densities and included embedded test carbon steel coupons (SI sections 1.1 and 1.2 and Supplementary Figures 1, 2). The MX-80 bentonite was used in its granular form or as pre-compacted blocks. The modules (SI sections 1.3 and 1.4) were, at 14°C, submerged into an anoxic porewater-filled borehole (named BIC-A) within the OPA at the Mont Terri underground rock laboratory in St-Ursanne, Switzerland, and remained in the borehole for variable durations: approximately 1, 1.5, 2.5, or 5.5 years (SI sections 1.4 and 1.5 and Supplementary Figure 3 top). The 1.5-year microbial cultivation results were previously published (Smart et al., 2017). It should be noted that changes in the experimental details of the preparation of the modules deployed for 2.5 years precluded direct comparison with other years. Thus, only the 1-, 1.5- (Smart et al., 2017), and 5.5-year block modules were selected for a comparison of enumerated viable heterotrophic aerobes, anaerobes, and SRB (SI sections 1.2 and 1.3). Cultivation-independent 16S rRNA gene V4-region microbiome profiling was performed and is presented for the 1- and 5.5-year modules.

### Enumeration and 16S rRNA Amplicon Sequencing

Borehole porewater was sampled for chemical analysis and DNA microbiome profiling (SI sections 1.6 and 1.7 and Supplementary Tables 1, 2). Post-retrieval, bentonite samples were collected in an anoxic glove box and kept at 4°C for enumeration and −20°C for gDNA extraction. Details of the bentonite cylinder sampling procedure are shown in SI section 1.5 for module samples and SI sections 1.8 and 1.9 for bentonite (Supplementary Figure 4) and swab samples. Heterotrophic aerobes and anaerobes were enumerated in triplicate from anoxically prepared bentonite suspensions, using the pour plate method of semi-solid R2A medium (Reasoner and Geldreich, 1985; Supplementary Table 3), cooled down to 45–50°C and grown under oxic (for 3 days) or anoxic (for 3 weeks) conditions at 30°C. Enumeration of SRB was performed with the most probable number (MPN) method (Hamon, 2003). Hungate tubes were filled with 9 ml of sterile, anoxic Postgate’s Medium B (Hamon, 2007; DSMZ, 2018) (Supplementary Table 4) and serial dilutions from 10^–1^ to 10^–5^ were made (in triplicates) using the same inoculum as for heterotrophic anaerobes and aerobes. The tubes were sealed to maintain anoxic conditions during 7 weeks of incubation at 30°C (see SI section 1.8 for more details). A sampling of bentonite is described in SI section 1.8, and gDNA extraction from bentonite and forensic swab samples (Sarstedt AG & Co., KG, Nümbrecht, Germany) was performed according to the recommendation by Engel et al. (2019b,a) utilizing a modified protocol of the DNeasy PowerSoil Kit and DNeasy PowerMax Soil Kit (QIAGEN NV, Venlo, The Netherlands) (SI sections 1.9 and 1.10). The 16S rRNA V4 region gene amplicons (total samples, *n* = 377) were prepared using the Quick 16S NGS Library Prep Kit (Zymo Research, Irvine, United States) and sequenced at the Lausanne Genomic Technologies Facility (University of Lausanne, Switzerland) using a MiSeq platform in a paired-end 300 bp mode (Illumina Inc., San Diego, United States). Details of the bioinformatics pipeline, based on USEARCH version 11 (Edgar, 2010), can be found in SI section 1.10. The R-package ampvis2 (Andersen et al., 2018) was used to analyze the dataset for similarities and explanatory variables.

### Bentonite Water Content, Dry Density, and Oxygen Detection

Bentonite water content and dry density were based on the wet weight, wet volume, and the dry weight after drying for 7 days at 105°C (SI section 1.11). The presence of molecular oxygen, trapped within 300 g of as-received bentonite, was determined by gas chromatography of the gas phase of bentonite isolated within gas-tight glass bottles (SI section 1.12). Gas analysis was performed using a Varian GC-450 (Agilent Technologies, Santa Clara, United States) equipped with a molecular sieve column (CP81071: 1.5m*1/8” UltiMetal MolSieve 13 9 80-100 mesh) and a thermal conductivity detector (TCD).

Additional details of the materials and methods used are available in SI section 1.

## Results

We considered the spatially resolved, 16S rRNA amplicon-based distribution of microorganisms around, on, and in stainless steel modules containing bentonite as a function of deployment time. The spatial distribution was presumed to be impacted by the geochemical conditions that vary from the outside going inwards: on the outside, the porewater imposes the geochemical conditions; next, the module and its water exchange holes provide a metal surface for porewater microorganisms; then, the sintered stainless steel filter (18 μm average pore size) is also in contact with porewater microorganisms and so is the very surface of the bentonite cylinder; inside the bentonite cylinder, the bulk bentonite was considered separately from the bentonite adjacent to the carbon steel coupons (Supplementary Figures 1, 2).

### The Microbiome in Opalinus Clay Porewater

The chemical composition of the BIC-A borehole water was assayed from 2013 to 2018 (Supplementary Table 5). The results indicate that it is consistently anoxic: reduced iron is detected most years (except 2017), suggesting either microbial iron reduction or the corrosion of the stainless steel filter with sulfide.

The porewater microbiome was characterized as a function of time. Before deployment of the experiment (in 2013), the borehole porewater microbiome (Bagnoud et al., 2016) was dominated by taxa from the Peptococcaceae family (∼68%) and by SRB (∼66%) (Supplementary Table 6), and two of the most abundant genera were *Desulfotomaculum* and *Desulfosporosinus*, both gram-positive SRB. Additionally, an OTU identified as *Pseudomonas* sp. represented approximately 26% of the community. About 1.5 years of post-deployment (July 2014), the porewater microbiome revealed a significant change in composition (Supplementary Table 6) epitomized by a large increase in the relative abundance of *Pseudomonas* to ∼87% and a stark decrease in SRB genera to ∼4%. In July 2017, 4.5 years into the experiment, the community changed again, as evidenced by the lower abundance of *Pseudomonas* (∼31%) and higher contribution of SRB (∼19%) (Supplementary Table 6). Finally, after 5.5 years (July 2018), the community seemed to have reached a steady state as its composition resembled that of the year before.

### The Microbiome on Surfaces in Contact With Porewater

**FIGURE 1:**
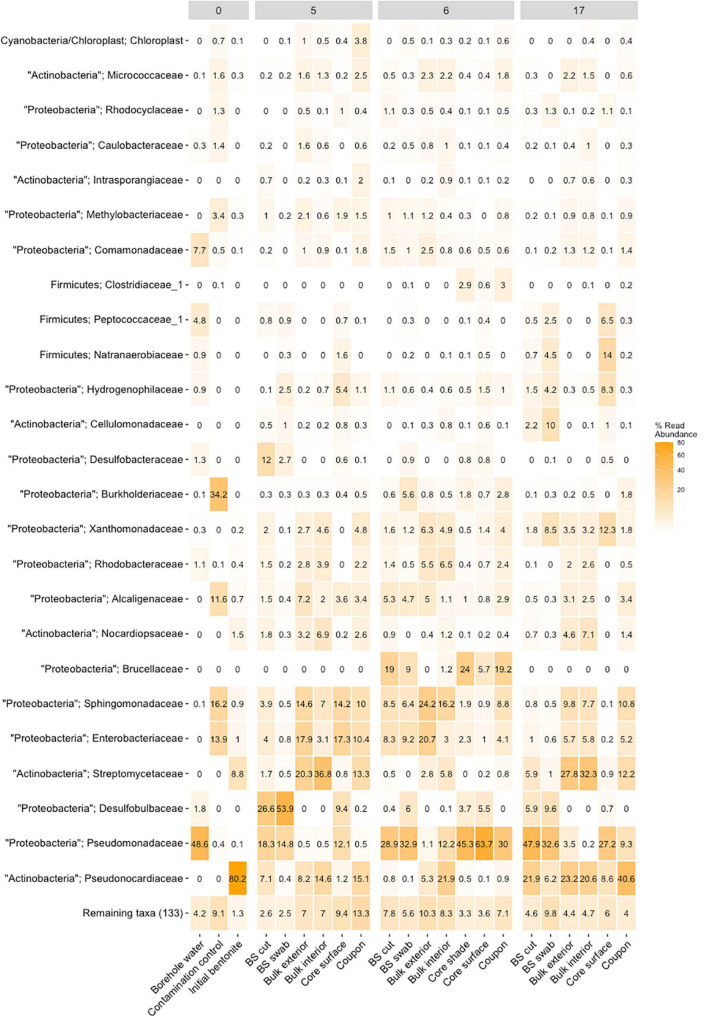
Heatmap of relative abundance of the top 25 detected main taxa (OTU) in the bentonite of three representative modules M5 (1.25 g/cm^3^, 5.5 years), M6 (1.55 g/cm^3^, 5.5 years), and M17 (1.25 g/cm^3^, 1 year), compared with the initial bentonite, the borehole water, and the contamination control denoted as 0. The modules are referred by their numbers at the top of the figure (e.g., 5 refers to module 5). For each of the bentonite samples from the three modules, the detected taxa are shown for the respective sampling sites, namely, black spot (BS) cut, black spot (BS) swab, bulk interior, bulk interior, core surface, and coupon. The remaining taxa, minor contributions, are combined within the “remaining taxa” group.

The module surface, the sintered stainless-steel filter, and the outer surface of the bentonite cylinder are all in contact with porewater. At early deployment times (1, 1.5, and 2.5 years), we observed isolated black spots on the surface of the bentonite cylinders (Supplementary Figures 5, 6). At the 5.5-year time point, the spots grew and the entirety of the cylinder surface was black (Supplementary Figure 7). Similarly, with longer deployment times, an increase in the black color of the stainless steel sintered filter was observed, suggesting that it was reacting (Duan et al., 2006), presumably with dissolved sulfide from the porewater. Furthermore, DNA-based microbiome profiling revealed that the porewater microbial community colonized the module surface, the water exchange holes (Supplementary Figure 2), and the sintered filter, as shown in Supplementary Figure 8. In fact, the microbiomes observed on these surfaces are dominated by the families Pseudomonadaceae, Peptococcaceae, Natranaerobiaceae, Desulfobulbaceae, and Desulfobacteriaceae, which are typically observed in the BIC-A borehole water (Supplementary Table 6). The bentonite cylinder surface, darker shades observed on that surface, and the aforementioned black spots are also shown to harbor a microbiome closely related to the borehole community (Figure 1 and Supplementary Figures 8, 9).

### Bulk Bentonite Microbiome

**FIGURE 2:**
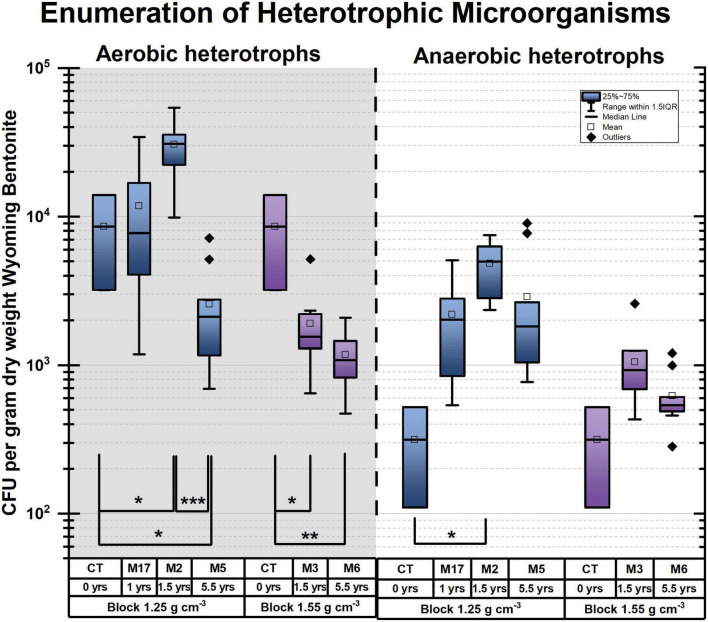
Colony-forming units (CFU) per gram dry weight (gdw) bentonite. (A) Absolute counts on a log scale, aerobic (gray background) and anaerobic heterotrophs (white background), box plots as a function of dry density and time. Brackets with * indicate significant *p*-values < 0.05, **p-values of < 0.005, ***p-values of < 0.0005 from pairwise Student’s *t*-test. Undeployed samples are labeled CT (bentonite powder-pellet mix used to produce blocks). M refers to the module. For instance, M2 refers to module 2.

**FIGURE 3:**
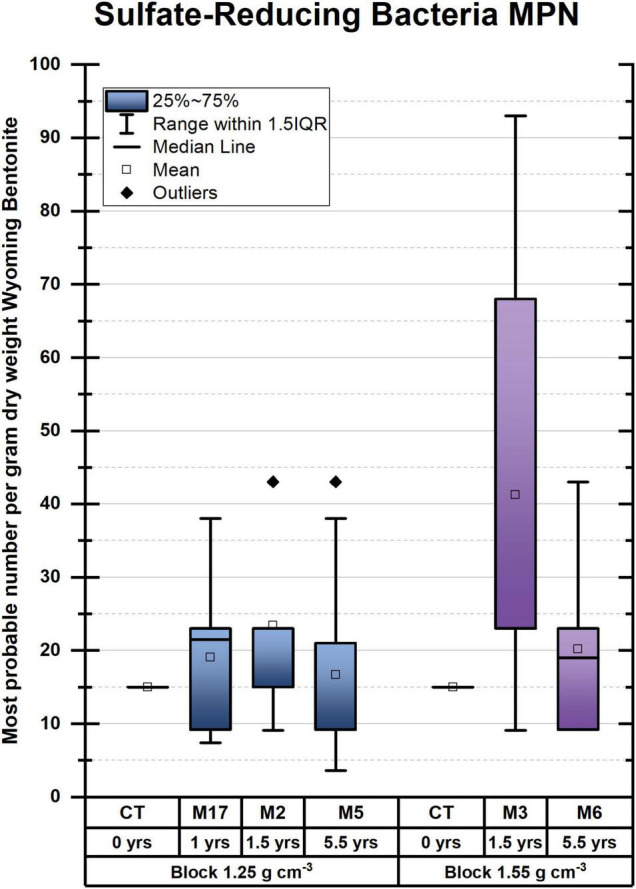
Sulfate-reducing bacteria enumerated as most probable number (MPN) per gram dry weight bentonite. No statistically relevant pairwise differences could be discerned. M refers to the module. For instance, M2 refers to module 2.

When assessing the microbiome based on DNA profiles (Figure 1 and Supplementary Figures 8, 9), one must recognize the stabilization of DNA on clay (Greaves and Wilson, 1969; Engel et al., 2019a), resulting in the inclusion of the signature of nonviable microbes. In addition to DNA-based analyses, the number of viable cells was enumerated as colony-forming units (CFU) over the entirety of each bentonite cylinder (*n* = 10 per module and *n* = 2 for undeployed bentonite) for anaerobic and aerobic heterotrophs (Figure 2) and using the MPN method for SRB (Figure 3). The water content of the deployed bentonite was also measured and is shown in SI (Supplementary Table 7).

In blocks with a dry density of 1.25 g/cm^3^, significant growth of both aerobic and anaerobic heterotrophs is observed when comparing undeployed bentonite (CT) to that deployed in the borehole for 1.5 years (M2) (Figure 2 and Table 1). However, the comparison of 5.5 years of deployment (M5) with the initial sample (CT) and with the deployment for 1.5 years (M2) reveals a significant decrease in the number of aerobic heterotrophs. This finding is confirmed when comparing aerobic counts for 1 year (M17), 1.5 years (M2), and 5.5 years (M5) using one-factor analysis of variance (ANOVA) (Table 1), indicating time as a significant explanatory variable. The anaerobic CFU per gram dry weight (CFU/gdw) counts decrease to a lesser extent than the aerobic counts between 1.5 and 5.5 years, and the change is not statistically significant based on the Student’s *t*-tests for 0–5.5 years (CT vs. M5, *p*-value of 0.266) or 1.5–5.5 years (M2 vs. M5, *p*-value of 0.090) (Table 1). Thus, for the lower density (1.25 g/cm^3^), the counts of both aerobes and anaerobes increase until 1.5 years but subsequently, aerobe counts decrease while anaerobe counts remain near constant until 5.5 years (Figure 2 and Table 1).

**TABLE 1:** Significance values for comparison of aerobic and anaerobic heterotrophs and SRB (Figure 1).

Deployment duration	Module number	Dry density	Analysis	*p value*		
				aerobes	anaerobes	SRB
1 year	M17	1.25 g/cm^3^	one-factor ANOVA:time	**3.4.10^–6^**	**0.028**	0.430
1.5 years	M2	1.25 g/cm^3^				
5.5 years	M5	1.25 g/cm^3^				
0 years	CT	1.25 g/cm^3^	Student’s *t*-test:time	**0.046**	**0.006**	nd
1.5 years	M2	1.25 g/cm^3^				
0 years	CT	1.25 g/cm^3^	Student’s *t*-test: time	**0.032**	0.266	nd
5.5 years	M5	1.25 g/cm^3^				
1.5 years	M2	1.25 g/cm^3^	Student’s *t*-test:time	**2.5.10^–6^**	0.090	0.246
5.5 years	M5	1.25 g/cm^3^				
0 years	CT	1.55 g/cm^3^	Student’s *t*-test:time	**0.009**	0.133	nd
1.5 years	M3	1.55 g/cm^3^				
0 years	CT	1.55 g/cm^3^	Student’s *t*-test time	**0.003**	0.182	nd
5.5 years	M6	1.55 g/cm^3^				
1.5 years	M3	1.55 g/cm^3^	Student’s *t*-test:time	0.110	0.056	0.081
5.5 years	M6	1.55 g/cm^3^				
Student’s *t*-test: aerobes CFU/gdw vs. anaerobes CFU/gdw			1.5 years	M3	1.55 g/cm^3^	0.067
			5.5 years	M6	1.55 g/cm^3^,	**0.010**

*Bold values denote statistical significance at the **p** < 0.05 level. nd, not defined due to insufficient number of observations.*

As for the higher density blocks (1.55 g/cm^3^), there is a significant decrease in the number of cultivable aerobes during the first 1.5 years (CT and M3) (Figure 2 and Table 1). However, it is followed by an insignificant change between 1.5 and 5.5 years (M3 and M6) (Figure 2 and Table 1), highlighting the persistence of aerobes. Furthermore, the number of cultivable anaerobes did not change significantly from deployment to 5.5 years (Figure 2 and Table 1). In summary, for the higher density, aerobes decrease in number until 1.5 years while anaerobes numbers do not change. Subsequently, both aerobes and anaerobes remain constant.

Finally, for both anaerobic and aerobic heterotrophs, there is no evidence for differences in the viable counts when considering the location of the bentonite bulk sample (*n* = 5 for each per module, data not shown). In other words, comparing bulk samples collected farther from or closer to the surface did not result in a difference in viable counts. This result hints at the fact that bulk bentonite microorganisms may originate from the sourced bentonite rather than from the borehole porewater.

**FIGURE 4:**
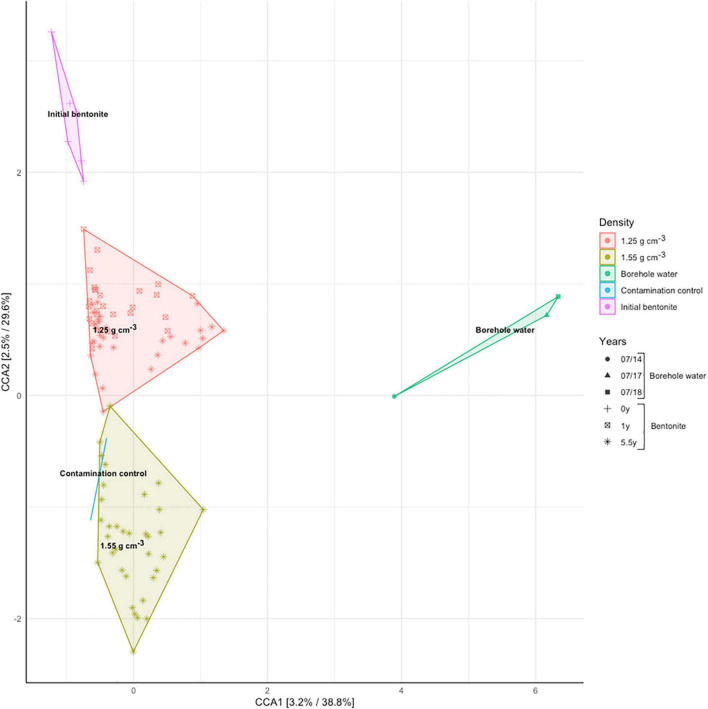
Canonical correspondence analysis (CCA, Hellinger-transformed data) of 16S rRNA gene amplicon data for modules M17 (1.25 g/cm^3^, 1 year), M5 (1.25 g/cm^3^, 5.5 years), and M6 (1.55 g/cm^3^, 5.5 years), as presented in Figure 1 and Supplementary Figure 9, constrained for the bentonite dry density as the explanatory variable. The time dependence is represented by the shape of the data markers (see legend).

Analysis of 16S rRNA gene amplicon sequences using a canonical correspondence analysis evidenced the fact that dry density shapes the bulk bentonite microbiome (Figure 4). The growth-related change in the microbial community upon saturation with porewater is revealed by the separation between the initial and the deployed bentonite samples and between the two dry densities considered.

For SRB, enumeration by the MPN method revealed very low numbers of viable cells counts compared with heterotrophs, in the range of 10–100 MPN/gdw bentonite for SRB (Figure 3) compared with bentonite for anaerobic heterotrophs up to ∼7,500 CFU/gdw (Figure 2). Among the deployed modules, the highest number of culturable SRB was found after 1.5 years in M3 despite the fact that the bentonite had the highest observed dry density (measured at 1.925 g/cm^3^ ± 0.2285 g/cm^3^ (STD), aimed for 1.55 g/cm^3^). Additionally, in contrast to heterotrophic anaerobes, SRB shows no increase in numbers between the initial bentonite and the first time point.

These results suggest that, in the case of 1.25 g/cm^3^, SRB does not grow during the initial 1.5 years, in contrast to what was observed for other anaerobic microorganisms and aerobes (Figure 2). Comparing SRB numbers within bulk bentonite as a function of distance from the bentonite cylinder surface, we could not observe any trend either (data not shown), suggesting no colonization of the bentonite bulk by borehole water SRB. Had such a colonization taken place, a gradient of SRB abundance would have been expected from the exterior to the interior of the bentonite bulk.

**FIGURE 5:**
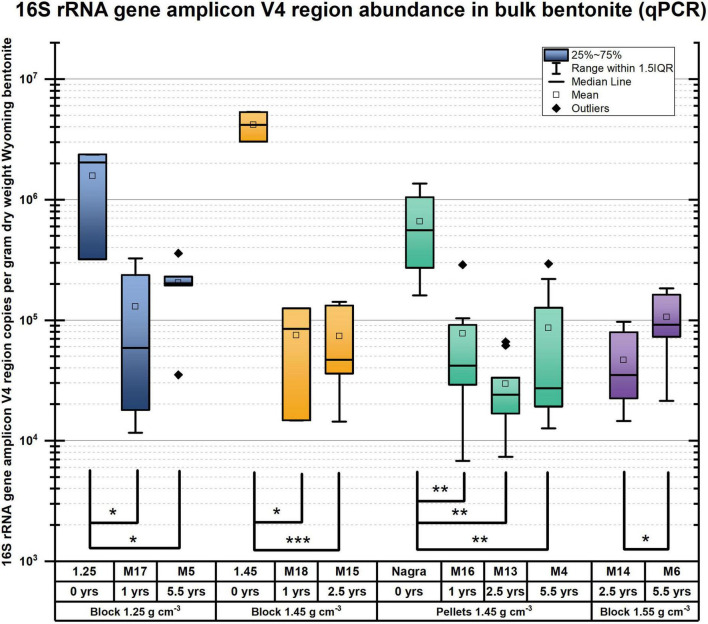
16S rRNA gene amplicon V4 region semiquantification in bulk bentonite samples from all initial block and pellet bentonite (denoted as Nagra) and 1-, 2.5-, and 5.5-year bentonite modules, represented as box plot, normalized to the dry weight of the bulk bentonite sampled. A significant change in the detectable 16S rRNA gene amplicon copy numbers is observed from all initial states, except for the 1.55 g/cm^3^ bentonite due to the absence of an initial time point sample. *p*-values from pairwise Student’s *t*-test are indicated by brackets, * *p* < 0.05, **p < 0.005, ***p < 0.0005. The only significant change after the bentonite is deployed is observed for the high density of 1.55 g/cm^3^ bentonite (purple) from the 2.5-year to 5.5-year deployed bentonite, showing increasing copy numbers of 16S rRNA genes for the longer deployment time.

A comparison of the 16S rRNA gene amplicon-based analysis of the microbiome within deployed bentonite cylinders (M17, M5, and M6) with that in initial (undeployed) bentonite was performed. Quantification of 16S rRNA gene copy numbers was performed with certain methodological limitations (see SI section 1.11 for more details). The quantification of the 16S rRNA genes shows a few variations after deployment (Figure 5), allowing comparison across time. The undeployed bentonite microbiome was observed as a community primarily composed of aerobic heterotrophs such as Streptomycetaceae, Pseudonocardiaceae, and Nocardiopsaceae (Figure 1 and Supplementary Figure 9). The community associated with samples from the bulk bentonite deployed for 1 year (bulk bentonite, coupon bentonite, coupon swab; M17, 1.25 g/cm^3^) reflects the growth of aerobes with major contributions by OTUs related to the three families present in the undeployed bentonite but also with the emergence of the families Xanthomonadaceae, Comamonadaceae, and Burkholderiaceae (Figure 1 and Supplementary Figure 9). Sphingomonadaceae, Enterobacteriaceae, and Alcaligenaceae were not considered as they were also detected in the negative control. Thus, despite the anoxic borehole environment, it is evident that aerobic microorganisms were able to grow in the 1.25 g/cm^3^ density bentonite during the first year. For the 5.5-year time point (M5), we observed that the microbiome has changed, but the contribution of aerobes does not appear to have decreased and no SRB is detected among the major contributing OTUs in the bulk bentonite (Figure 1 and Supplementary Figure 9).

**FIGURE 6:**
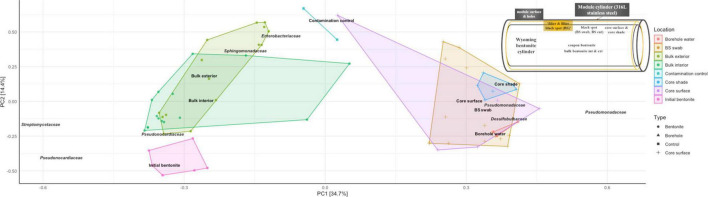
Unconstrained principal component analysis (PCA, Hellinger-transformed data) of 16S rRNA gene amplicon data for modules M17 (1.25 g/cm^3^, 1 year), M5 (1.25 g/cm^3^, 5.5 years), and M6 (1.55 g/cm^3^, 5.5 years), the initial bentonite, the borehole water, and the contamination control as presented in Figure 1 and Supplementary Figure 9. The samples are color-coded according to their sampling location whose approximate location is depicted in a sketch on the top right: initial bentonite in pink, bulk bentonite in green, core surface in purple and blue, black spots in orange, and borehole water in red. BS swab represents black spot swab sample; core shade represents discolored bentonite cylinder surface.

Spatial differences in the microbiome were observed along the borehole porewater-bulk bentonite axis for all sampled timeframes and densities. This general trend is exemplified in an unconstrained principal component analysis (PCA) of the 16S rRNA gene amplicon data (Figure 6). It shows the segregation of the microbiomes according to their location along the cross-sectional gradient extending from the borehole water to the bulk bentonite. The OTU clustering occurred along three axes: (i) the aerobic Pseudonocardiaceae and Streptomycetaceae; (ii) aerobic and anaerobic Enterobacteriaceae and Sphingomonadaceae; and (iii) borehole-derived anaerobic Pseudomonadaceae and Desulfobulbaceae. Evident is the divide between the initial and deployed bentonite microbiomes. More importantly, bulk bentonite samples cluster more closely with the initial bentonite than the samples obtained from the bentonite core surface or the observed black spots. Furthermore, bentonite cylinder surface samples, including black spot samples, cluster more closely with the borehole water samples, characterized by OTUs related to Pseudomonadaceae and Desulfobulbaceae, than with the bulk bentonite. This relationship further supports the exclusive bentonite origin of the bulk bentonite community.

## Discussion

### Opalinus Clay Porewater Microbiome

The chemical signature of the borehole porewater suggests that anoxic conditions were maintained. Sulfide was only detectable in the 2013 sample, suggesting activity of SRB, which is consistent with the high concentration of sulfate (15.6–23.3 mM). Its lack of detection in other years could be due to its precipitation with iron. An abrupt change in the microbiome composition was observed in 2014, showing *Pseudomonas* as the major genus and the functional group of SRB genera at only 4%. The reasons for this change are unknown but could be due to the disturbance associated with the deployment of the experiment in the borehole, e.g., organic carbon (representing 0.20–0.28% of the bentonite by weight) (Karnland, 2010) may potentially have been released from the bentonite placed within the modules and provided an ecological advantage to the fast-growing *Pseudomonas*.

### Bulk Bentonite Microbiome

DNA sequencing, combined with the cultivation data, suggests that there were growth and change in the microbiome composition over time. Additionally, the distinct distribution of the low- and high-density samples indicates that the differentiation in the microbial community is associated with the dry density. In essence, while starting from an identical initial inoculum, the communities develop in different directions at the two densities. Furthermore, the fact that the two deployed bulk bentonite communities are vastly different from the borehole community point to the inability of the borehole water microbiome to colonize the bulk bentonite within the sampled timeframe. Finally, the clustering of samples from the low density of 1.25 g/cm^3^ and 1-year deployment (M17) with those of the 5.5-year deployment (M5) (Figure 4) indicate a temporally stable bentonite community.

Thus, provided there is sufficient pore space available (i.e., a low dry density), the number of viable heterotrophs increases up until 1.5 years but decreases or stagnates at longer deployment times. As expected and previously reported (Stroes-Gascoyne et al., 2010, 2013; Smart et al., 2017), the 1.25 g/cm^3^ of bentonite density appears to create better conditions for microbial life than the 1.55 g/cm^3^ of density. This high dry density appears to inhibit the growth of aerobes and limit their viability. However, the number of viable aerobes remained stable after 1.5 years, despite extended exposure to an anoxic environment. Anaerobic heterotrophs coped better with the high dry density (1.55 g/cm^3^) than aerobic heterotrophs, showing CFU/gdw numbers comparable with the initial state after deployments of either 1.5 or 5.5 years. Additionally, we observed that the number of cultivated aerobes always exceeds that of cultivated anaerobes (Figure 2). Thus, the persistence of aerobic heterotrophs cannot be attributed solely to facultative anaerobes but indicates the persistence of obligate aerobes. For example, at 5.5 years, for 1.55 g/cm^3^, the number of aerobic heterotrophic CFUs is significantly larger than anaerobic heterotrophic CFUs, despite 5.5 years of exposure to an anoxic environment (Table 1).

The observations regarding sulfate-reducing bacteria lead to the conclusion that the few SRB found in the bentonite cylinders are likely to be bentonite-derived. While present, they remain inactive, as no growth was observed as a function of deployment time. Possible explanations for the absence of porewater-derived SRB are that (a) the bentonite has swelled sufficiently from pre-deployment saturation to preclude the inward migration of porewater microbes once deployed in the borehole, (b) the conditions for the growth of porewater microbes are too challenging within the bentonite, or (c) both.

The DNA sequencing-based identification of an aerobic microbiome and the absence of SRB among the major OTUs in bulk bentonite confirm the enumeration results showing a viable aerobic community and the muted presence of SRB. Furthermore, the taxa detected near the coupons (Figure 1 and Supplementary Figure 9) appear to be governed by the surrounding bentonite, i.e., the proximity of metallic coupons had no discernable effect on the bentonite community.

DNA quantification from bentonite gave only limited insight into the abundance of microbial life because many quantification attempts failed due to the very low biomass and thus, low starting DNA material. A significant decline in 16S rRNA gene copy numbers can be observed from the initial bentonite samples for the lowest dry density blocks (1.25 g/cm^3^) when compared with the modules deployed for 1 year (M17, *p* = 0.022) and 5.5 years (M5, *p* = 0.026) (Figure 5). Similarly, for the medium dry density, a significant decline in 16S rRNA gene copy numbers was observed when comparing the initial sample (1.45 g/cm^3^, yellow) with the modules deployed for 1 year (M18, yellow, *p* = 0.017) and 2.5 years (M15, yellow, *p* = 5⋅10^–6^). A significant increase of the number of 16S rRNA gene copy numbers was observed from the 2.5-year to 5.5-year modules for 1.55 g/cm^3^ blocks (M14&M6, purple, *p* = 0.041) but not for 1.45 g/cm^3^ of pellet bentonite modules from 2.5-year to 5.5-year modules (M13&M4, green, *p* = 0.080). Also, for the lowest dry density block (blue), no significant difference could be discerned when comparing 1-year with 5.5-year 16S rRNA gene amplicon copies (M17&M5, *p* = 0.393). DNA amplification showed a decrease of 16S rRNA gene amplicon copies from the initial, unexposed bentonite, compared with the 1-year block and pellet modules for all dry densities except 1.55 g/cm^3^ (Figure 5). This is in contrast with the enumeration of anaerobic and aerobic heterotrophs, which were shown to increase (see Figure 1). However, the abundance of the 16S rRNA gene amplicons per gram dry weight appears relatively stable over all sampled modules (1, 2.5, and 5.5 years), which is not surprising as the detection of DNA does not allow conclusions about microbial viability. For example, dead bacteria cells lyse, liberating their DNA which then can be adsorbed by clay minerals in bentonite and stabilized over long timeframes, allowing their signature to be detected using a molecular biology analysis (Greaves and Wilson, 1969; Engel et al., 2019a). Thus, we hypothesize that the initial time point includes substantial DNA from non-viable cells.

### Persistence of Aerobes

Notably, the number of microorganisms grown from bulk bentonite under oxic conditions exceeded that grown under anoxic conditions. This suggests that there were at least a fraction of the aerobic microorganisms that were strict aerobes. The growth of strict aerobes under anoxic conditions up until 1.5 years (for the low-density condition) and their persistence for up to 5.5 years (for both densities) suggests that oxygen may also persist to some extent in the bentonite. We hypothesize that oxygen adsorbed to the clay mineral surfaces may facilitate the survival of aerobes. Based on the work of Nakayama (Nakayama, 1958) on O_2_ sorption to bentonite, we calculated the amount of bentonite-associated oxygen for each module: 1.32–5.78 mmoles (1.25 g/cm^3^, 2,356 g total dry-weight bentonite), 1.53–6.71 mmoles (1.45 g/cm^3^, 2,733 g total dry-weight bentonite), and 1.63–7.17 mmoles (1.55 g/cm^3^, 2,921.75 g total dry-weight bentonite). The range of values results from the range of two moisture measurements of the as-received bentonite, 6.4 and 9.8% (w/w). We hypothesize that the adsorbed oxygen is mobilized, and thus bioavailable, upon saturation of the bentonite, due to the increased spacing of the montmorillonite interlayer that is facilitated by hydration, by analogy to the observation reported for H_2_ (Edge et al., 2014). Nevertheless, this mobilization of oxygen does not appear to affect the corrosion of metallic coupons as evidenced by the measured rates and the chemistry of corrosion products (Smart et al., 2017; Reddy et al., 2021). It also does not appear to significantly affect the chemistry of the borehole water which has remained strictly anoxic.

The fact that growth is observed for heterotrophic anaerobes (perhaps facultative anaerobes) but not for SRB bolsters the case of delayed onset of SRB growth in bentonite due to the presence of bioavailable oxygen. A desorption test of ambient-humidity bentonite at room temperature was performed and reported detectable oxygen ( > 0.05 vol.%) within the gas phase at around 7–9 days after isolation in a nitrogen atmosphere (Supplementary Figure 10 and Supplementary Table 8). This result suggests that SRB are inhibited due to the presence of potentially bioavailable oxygen. As oxygen is expected to be consumed over time, the potential for an increase in the relative abundance of SRB in bulk bentonite for longer deployment times remains. If that were the case, an increase of SRB in the bulk bentonite at least within the 1.25 g/cm^3^ module would be expected at future time points.

### Carbon Steel Corrosion

The rate of corrosion of carbon steel test coupons embedded in bentonite was investigated for the 1. 5-, 2. 5-, and 5.5-year time points for the high-density (1.55 g/cm^3^) blocks (Smart et al., 2017; Reddy et al., 2021). The corrosion rate of coupons was shown to decrease for longer deployment times, presumably due to surface protection by corrosion products. Thus, for bentonite blocks (1.55 g/cm^3^), the corrosion rate was reported to reach up to ∼1.5 μm/year after ∼1.5 years and to decrease to ∼1.1 μm/year after ∼2.5 years and to ∼0.75 μm/year after ∼5.5 years. Hence, the corrosion rates appear to evolve as they would in the absence of microorganisms. This is consistent with the lack of colonization of bulk bentonite by SRB, which is expected to be the main metabolic group responsible for MIC under anoxic conditions.

## Conclusion

Overall, we conclude that for partially pre-saturated bentonite, further saturation in the borehole is accompanied by the growth of a heterotrophic microbiome and that bioavailable oxygen remains trapped with the bentonite (e.g., *via* adsorption) for a long period of time (i.e., years). These findings reshape our understanding of the predominant microbial processes upon repository gallery closure. Microbes will increase in numbers until full saturation is reached and will remain at stable numbers over extended periods (e.g., decade(s)). Furthermore, the main source of microorganisms in the bentonite appears to be the bentonite itself rather than the host rock or its porewater. In fact, the data do not support the possibility for borehole water microorganisms, including SRB, to colonize the bentonite. We can further conclude that the combination of cultivation-based methods and molecular tools provides valuable insights into the growth and persistence of microorganisms in swelling, unsaturated bentonite clay. The observed growth of aerobic heterotrophs and the suppression of SRB can be expected in all nominally anoxic environments in which Wyoming bentonite is deployed, e.g., in the case of the abovementioned bentonite experiment in granite host rock (Engel et al., 2019b). Finally, there is currently no direct evidence of sulfate reduction within bentonite or of MIC of the carbon steel coupons.

## Data Availability Statement

The datasets presented in this study can be found as Zenodo repository and includes raw data of cultivation and raw DNA sequences under the doi: 10.5281/zenodo.4883717.

## Author Contributions

RB-L and NB wrote the manuscript. ND and RB-L planned the experiment. RM, MF, BR, AR, and NB performed the experimental work. RB-L, AJ, and NB performed the data analysis. All authors have given their approval for the final version of the manuscript.

## Conflict of Interest

BR and AR were employed by Jacobs Engineering Group Inc. The remaining authors declare that the research was conducted in the absence of any commercial or financial relationships that could be construed as a potential conflict of interest.

## Publisher’s Note

All claims expressed in this article are solely those of the authors and do not necessarily represent those of their affiliated organizations, or those of the publisher, the editors and the reviewers. Any product that may be evaluated in this article, or claim that may be made by its manufacturer, is not guaranteed or endorsed by the publisher.
