# Supplementary material for: Growth and Persistence of an Aerobic Microbial Community in Wyoming Bentonite MX-80 Despite Anoxic in situ Conditions
Source: Front Microbiol. 2022 Apr 25;13:858324. doi: 10.3389/fmicb.2022.858324 (PMC9082992; doi:10.3389/fmicb.2022.858324)
Supplement: Supplementary file 1 [file Data_Sheet_1.docx]

Supplementary Material

Supplementary data and figures for “Growth and persistence of an aerobic microbial community in Wyoming bentonite MX-80 despite anoxic *in situ* conditions”

^a^Niels Burzan, ^a^Roberta Murad Lima, ^a^Manon Frutschi, ^b^Andrew Janowczyk, ^c^Bharti Reddy, ^c^Andrew Rance, ^d^Nikitas Diomidis, ^a^Rizlan Bernier-Latmani*

^a^Environmental Microbiology Laboratory, École Polytechnique Fédérale de Lausanne, Lausanne, Switzerland

^b^Bioinformatics Core Facility, Swiss Institute of Bioinformatics, Lausanne, Switzerland

^c^Jacobs Engineering Group Inc., Critical Missions Solutions, Harwell Science and Innovation Campus, Didcot, Oxfordshire, England, United Kingdom

^d^National Cooperative for the Disposal of Radioactive Waste, Wettingen, Switzerland

Table of Contents

[1 Materials & Methods 3](#_Toc96877757)

[1.1 Experimental setup 3](#_Toc96877758)

[1.2 Bentonite preparation 7](#_Toc96877759)

[1.3 Assembly of corrosion test modules 7](#_Toc96877760)

[1.4 *In situ* exposure 8](#_Toc96877761)

[1.5 Removal of test modules 9](#_Toc96877762)

[1.6 Porewater analysis 9](#_Toc96877763)

[1.7 DNA amplification from borehole porewater 10](#_Toc96877836)

[1.8 Bentonite sampling and microbial cultivation analyses 12](#_Toc96877848)

[1.9 DNA extraction from bentonite modules and swab samples 14](#_Toc96877849)

[1.10 DNA amplification from bentonite and module samples 15](#_Toc96877922)

[1.11 Water content, CFU & MPN per gram dry weight, observed dry density of bentonite 16](#_Toc96877923)

[1.12 Oxygen desorption from Wyoming bentonite 17](#_Toc96877924)

[2 Results 18](#_Toc96877925)

[2.1 Borehole water composition and microbiome 18](#_Toc96877926)

[2.2 Bentonite properties, cultivation of (an)-aerobic heterotrophs and sulfate-reducing bacteria and 16S rRNA gene semi-quantification 20](#_Toc96877927)

[2.3 16S rRNA gene amplicon sequence analysis 26](#_Toc96877928)

[2.4 Oxygen desorption from Wyoming bentonite 28](#_Toc96877929)

[3 Download link for NAGRA report and figure data 30](#_Toc96877930)

[4 APPENDICES 33](#_Toc96877931)

[4.1 Alpha diversity data, Shannon and Simpson 33](#_Toc96877932)

[4.2 Alpha Diversity plots 43](#_Toc96877933)

# Materials & Methods

## Experimental setup

Stainless steel modules, containing carbon steel and copper coupons (20 mm diameter, thickness of 10 mm for the former and 3 mm for the latter) embedded in Wyoming bentonite at three different densities (1.25, 1.45 or 1.55 g/cm^3^) and two different formulations (pre-compacted blocks or granular) were submerged in an anoxic, natural porewater-filled borehole within Opalinus Clay rock (**Figure S1**). This low-temperature (14ºC), anoxic and saline environment is expected to resemble the long-term conditions of a repository to be built into Opalinus Clay rock. The stainless-steel modules are designed to allow the free exchange of water with the borehole water (**Figure S2**). The microbial community of the porewater at the beginning of the experiment was previously analyzed by Bagnoud *et al*. ^1^

At the onset of the experiment (Jan. 2013), a set of 6 modules was deployed in the borehole (modules M1 – M6) (**Figure S3** top). After 1.5 years (Sept. 2014), 3 modules (M1-M3) were removed and the results of the corrosion and microbial cultivation published ^2^.The 3 modules removed at 1.5 years were replaced with a second set of modules (modules M13-M15) and exposed for 2.5 years (from Sept. 2014 to July 2017). However, it is important to note that, for modules M13-M15, the deployment started around 1.5 years into the overall experiment, not at the same time as the modules removed at 1.5 years (modules M1-M3). After removal of that second set (modules M13-M15), a third set of modules (M16-M18) was deployed for 1 year (from July 2017 to July 2018). Again, their exposure began not at the onset of the experiment but rather around 4 years into the overall experiment. In July 2018, that third set of modules (M16-M18), deployed for 1 year, and a subset of the first set of modules (M4-M6), deployed for 5.5 years (Jan 2013 to July 2018), were removed for analysis.


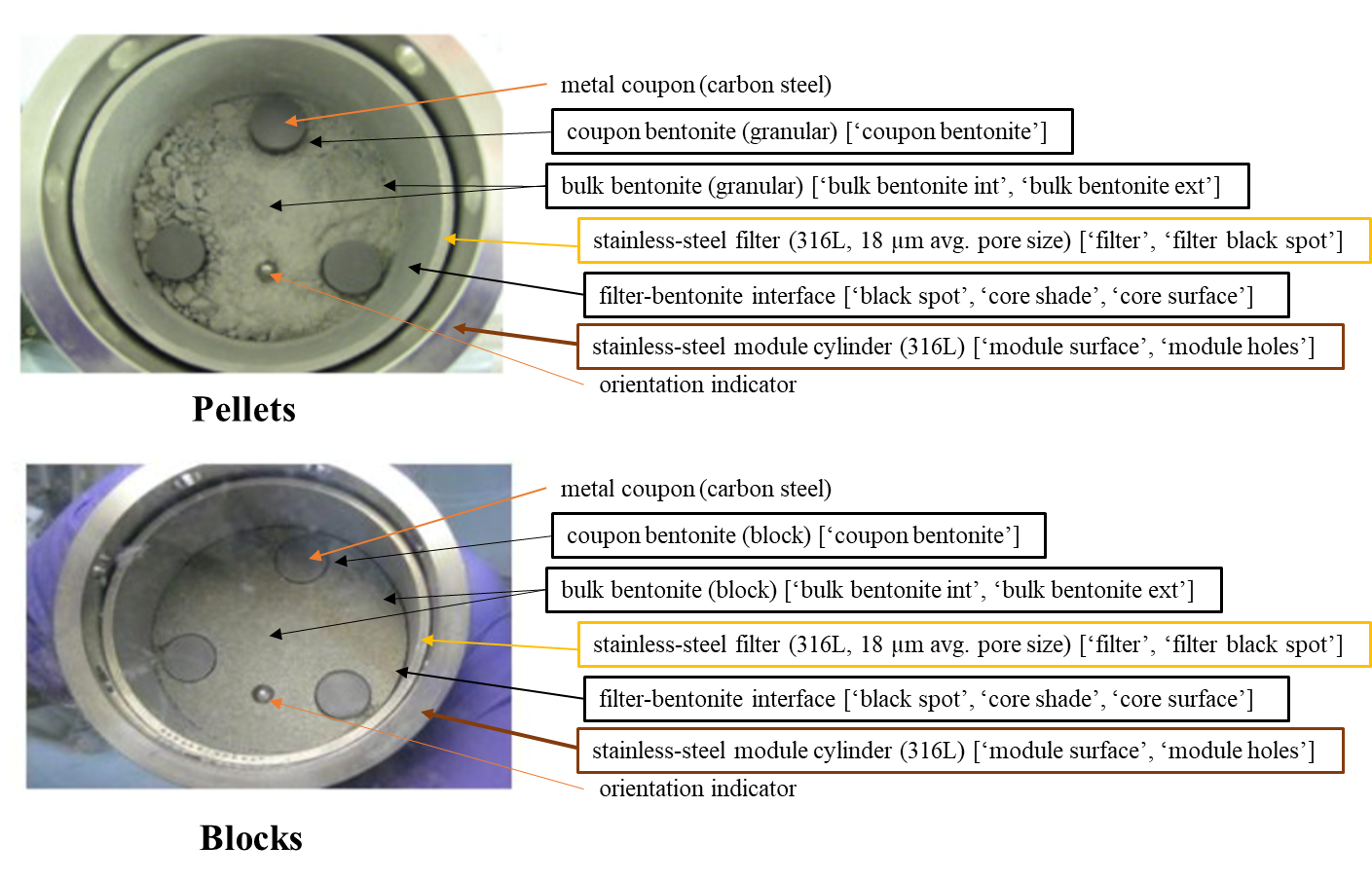


Figure S1: Illustration of the component of an experimental module along with the two types of bentonite formulations. The module is composed of three concentric cylinders. The outer-most is a stainless-steel cylinder (25cm long, 12.6 cm outer diameter) and contains a sintered stainless-steel filter that itself contains a solid cylinder of Wyoming bentonite, either as granular pellets or as pre-compacted blocks. In addition, sample site naming is indicated here: in black boxes for Wyoming bentonite samples, in the yellow box for stainless steel filter samples and in the brown box for module cylinder samples.


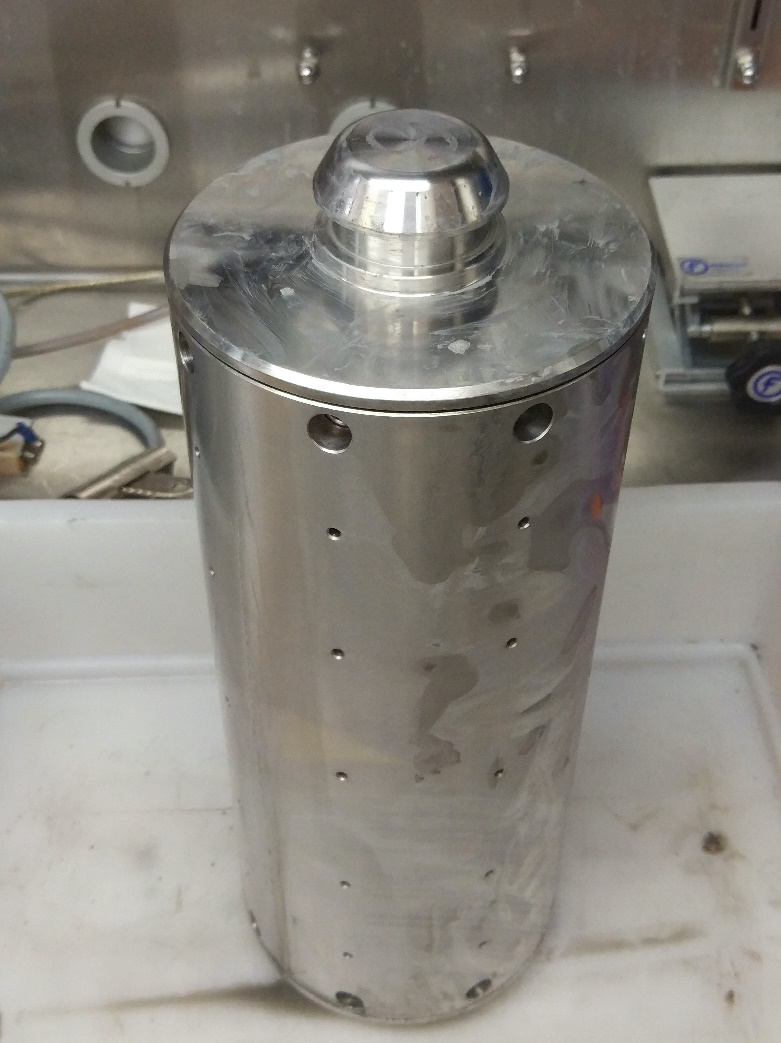


Water-exchange holes

Figure S2: Stainless-steel experimental module with water exchange holes and attached top lid (left photograph), filled with bentonite and embedded corrosion test coupons (right, rendering obtained from Smart et al. ^2^)


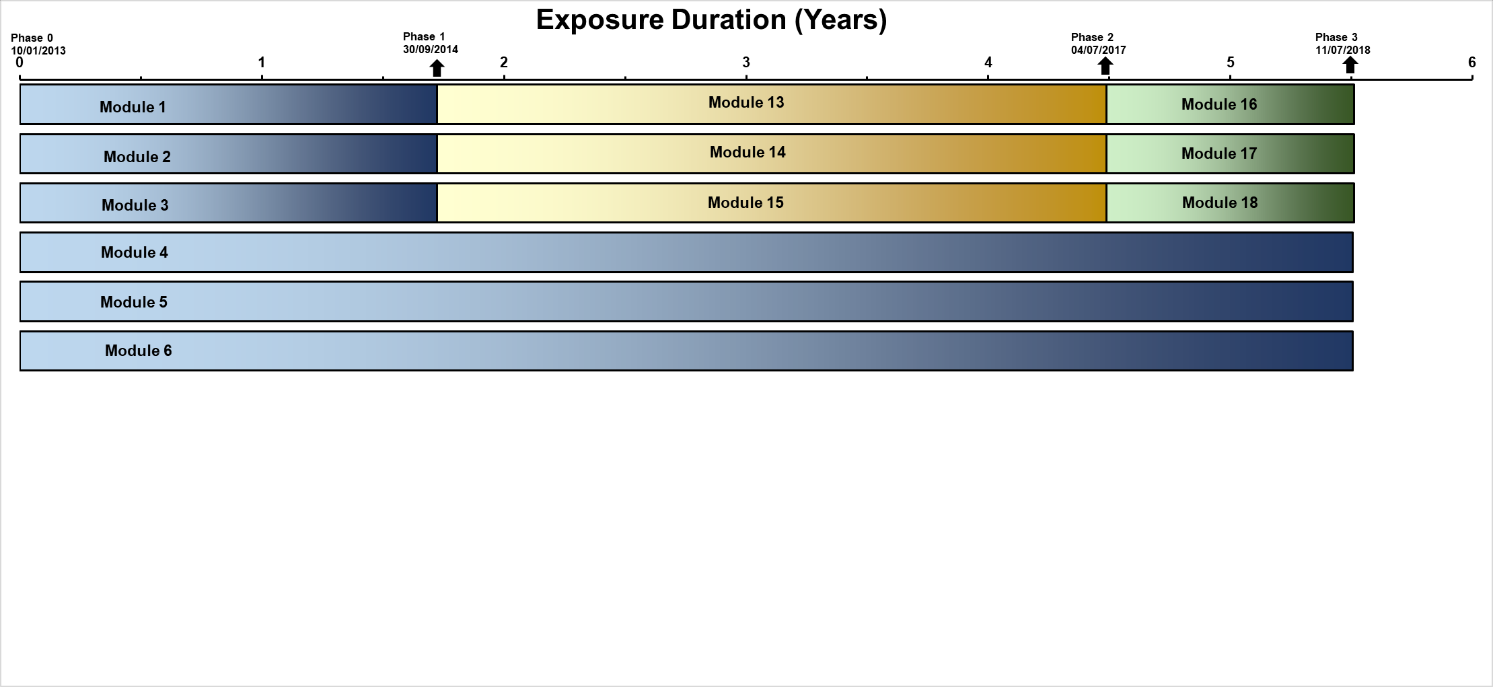


| module ID | dry density [g/cm^3^] | formu-lation | duration deployed | deployed in | Removed in | Analyses performed | Assembly conditions, comments | Cultivation data included in present paper? |
| --- | --- | --- | --- | --- | --- | --- | --- | --- |
| M1 | 1.45 | granular | 1.5 y | Jan 2013 | Sep 2014 | DD,  cultivation | oxic assembly, previously published^2^ | No, due to low number of samples |
| M2 | 1.25 | block |  |  |  |  | anoxic assembly, previously published^2^ | Yes |
| M3 | 1.55 |  |  |  |  |  |  |  |
| M4 | 1.45 | granular | 5.5 y |  | Jul 2018 | DD,  cultivation,  amplicon sequencing | oxic assembly | No, due to low number of samples |
| M5 | 1.25 | block |  |  |  |  | anoxic assembly | Yes |
| M6 | 1.55 |  |  |  |  |  |  |  |
| M13 | 1.45 | granular | 2.5 y | Sep 2014 | Jul 2017 |  | 5-d anoxic storage & anoxic assembly | No, due to different preparation relative to M1 and M4 |
| M14 | 1.55 | block |  |  |  |  | 18-month anoxic storage & anoxic assembly | No, due to different preparation relative to M3, M6, M17 and M18 |
| M15 | 1.45 |  |  |  |  |  |  |  |
| M16 | 1.45 | granular | 1.0 y | Jul 2017 | Jul 2018 |  | 5-d anoxic storage & anoxic assembly | No, due to different preparation relative to M1 and M4 |
| M17 | 1.25 | block |  |  |  |  | anoxic assembly | Yes |
| M18 | 1.45 |  |  |  |  |  | anoxic assembly | No, due to low number of samples |

Figure S3: (Top) Schematic of the deployment and retrieval schedule for the modules. (Bottom) Table summarizing the characteristics of the modules used in this study. Cultivation-based enumeration of microbes was performed for all modules, DNA extraction and analysis were performed for modules M4-M6, M13-M15, and M16-M18. DD= dry density measurement; amplicon sequencing= 16S rRNA V4 region amplicon sequencing; cultivation= cultivation of heterotrophs (aerobic and anaerobic) and SRB. Exclusion due to low number of samples refers to the fact that, in some cases, statistical analysis was not possible due to the low number of samples prepared under comparable conditions.

## Bentonite preparation

Corrosion coupons and preparation of modules filled with bentonite was carried out by the commercial laboratories of Jacobs Engineering Group Inc, located in Harwell, Oxfordshire, United Kingdom, in the same way as previously described ^2^. Bentonite was sourced from Wyoming, United States of America, and purchased as Volclay® MX-80 from Minerals Technologies, New York, U.S.A. Compaction into pre-formed blocks of 100 mm diameter of bentonite, maintaining space for the incorporation of the corrosion coupons, was performed by Clay Technology AB, Lund, Sweden, and the following dry-densities were produced: 1.25, 1.45 or 1.55 g/cm^3^. The compaction mold was flushed with nitrogen to prevent the trapping of excess air and the compaction of the bentonite was carried out while under vacuum at increased moisture. These measures were taken in order to minimize residual oxygen in the compacted bentonite. Bentonite blocks were sealed in Mylar bags within a nitrogen atmosphere and transported to Jacobs Engineering Group Inc in Harwell, Oxfordshire, United Kingdom. Upon arrival, the bentonite blocks were stored in an argon gas-filled glovebox until further processing. The assembly of the modules with blocks and corrosion coupons was performed in a controlled argon atmosphere. The second formulation, granular Wyoming bentonite, was provided at a dry density of 1.45 g/cm and stored under oxic conditions until assembly

## Assembly of corrosion test modules

The assembly of corrosion test modules was carried out by Jacobs Engineering Group Inc., Harwell, Oxfordshire, United Kingdom, as previously described ^2^. Stainless-steel modules with a length of 250 mm and an outer diameter of 126 mm were fabricated. A sintered stainless-steel filter (outer diameter of 106 mm with a wall thickness of 3 mm, porosity of 30% with average pore size of 18 μm) was inserted as a liner to prevent bentonite leakage through the water-exchange holes of the module. Prior to assembly, all parts were cleaned with acetone and deionized water. All bentonite block-bearing modules were assembled in the absence of oxygen, within an argon-filled glovebox. However, for granular bentonite-bearing modules, varying protocols were utilized for module assembly, depending on the batch. For the first set of modules (specifically modules M1 and M4) the modules were assembled in an air atmosphere.

For each module, four layers of 3 coupons were positioned within the bentonite (see Smart *et* *al*. ^2^ for details). The coupons were 5 mm away from the outer circumference of the bentonite and positioned at 120° angles. After assembly, all modules were partially pre-saturated with non-sterile, anoxic artificial Opalinus Clay porewater for two days (bentonite blocks) or ten days (granular bentonite) within an argon-filled glovebox. Because the first set of granular bentonite -bearing modules were assembled outside the glovebox, they were transferred into the glovebox for pre-saturation, with four cycles of vacuum and argon flushing applied to the assembled modules prior to being placed into the pre-saturation solution (artificial Opalinus Clay porewater[[1]](#footnote-1)) until approximately 80% saturation was reached. The prepared and pre-saturated modules were packed in three layers of Mylar™ to exclude oxygen exposure during shipping to the Mont Terri Underground Rock Laboratory (URL).

Starting with the second set of modules, M13, M14 and M15, all modules were assembled within the glovebox. For this, the granular bentonite was weighed under oxic conditions, transferred to the glovebox with four cycles of vacuum and argon flushing and left to equilibrate for 3 to 5 days within the glovebox prior to module assembly. Modules M14 and M15, which contained bentonite blocks, were stored for 18 months within the argon-gas filled glovebox. Pre-saturation and packing of the module were carried out as described for the first set of modules.

Assembly of the third set of modules, M16, M17 and M18, was more similar to the first set of modules: Bentonite blocks were produced by Clay Technology AB immediately before module assembly within the glovebox, i.e., no interim storage of blocks under anoxic conditions. However, the granular bentonite modules were prepared in the same manner as the second set of modules: bentonite was weighed in air and placed within the glovebox for 3-5 days. Pre-saturation and packing of the modules were performed in the same way as for previous sets of modules.

The difference in preparation of the three sets of modules resulted in a decrease in the number of modules suitable for comparative analysis of the impact of deployment time, dry density and bentonite formulation on the microbial community. First, we eliminated the complete second set of modules (M13-M15) due to the 18-month-long anoxic storage of bentonite blocks and the different granular bentonite preparation (3-to-5-day anoxic storage). Thus, among the pre-compacted blocks, the low dry density of 1.25 g/cm^3^ and high dry density of 1.55 g/cm^3^ resulted in a comparable subset with modules with varying deployment times. However, for the 1.45 g/cm^3^ blocks, a single block bearing module remained (for one time point), and was thus excluded. The granular formulation was eliminated as well, due to the changes in the module preparation for the second and third sets of modules. Their exclusion, in addition to the aforementioned elimination of the 2.5-year timepoint, lead to a total number of samples for a low number of time points. Thus, no reliable statistical comparison of the bentonite formulation (granular vs. precompacted block bentonite) could be performed.

## *In situ* exposure

The modules were deployed in a 15 m deep borehole in Opalinus Clay rock at the URL Mont Terri, St-Ursanne, Switzerland. The borehole was drilled in March 2012, flushed with argon gas four times and sealed with a hydraulic packer. Additional argon gas was supplied until an overpressure of 3.5 bars was achieved (to maintain anoxic conditions). It was estimated that Opalinus Clay porewater filled the borehole at a rate of 44 mL per day. The first set of 6 modules was deployed on January 10^th^ 2013 and the natural porewater accumulated since drilling was sampled and analyzed. An additional 9 L of nonsterile anoxic artificial porewater ^1^ was added in order to submerge all modules completely.

## Removal of test modules

The first set of three test modules (modules M1-M3) was removed for analysis on 30 September 2014 after 20 months (~1.5 y) of exposure. The second set of three modules (modules M13-M15) was removed for analysis on 4 July 2017, after 33 months (~2.5 y). The third set of six modules (modules M16-M18 and modules M4-M6) was removed in July 2018 after respectively 12 and 66 months (1 y and 5.5 y) of exposure (**Figure S3**). During removal, porewater samples were obtained from the borehole for microbial community and chemical analysis. The modules were removed from the borehole while purging with 0.2 µm-filtered argon gas and forensic swab samples (Sarstedt AG & Co KG, Nümbrecht, Germany) were taken immediately from the steel canister’s outer surface and the module water exchange holes (for all modules except modules M1-M3). The modules were then placed into custom-built stainless-steel transfer flasks that were filled with water from the borehole and purged with argon before transport to the UK for analysis. In the UK, the modules were placed into a pre-cleaned and sterilized (wiped with a 70% isopropyl alcohol solution) argon-purged glove box for dismantling. Forensic swab samples were taken from the transport flasks before the modules were placed and when the modules were removed from the transport flasks such that potential contamination of the bentonite microbiome could be traced.

## Porewater analysis

Upon opening the borehole to retrieve the modules, the borehole porewater was sampled. A sterile 1-liter glass bottle was attached to an eight-meter-long aluminum rod (70% ethanol disinfected) and lowered into the borehole several times to obtain enough porewater for the following analyses. On-site, a Hach multimeter, HQ30D (Hach, Loveland, Colorado, U.S.A.) was used, equipped with electrodes MTC101 and PHC201 to measure pH and redox potential (Eh). Porewater was syringe-filtered through 0.2 µm membranes and samples were obtained for subsequent chemical analysis of sulfide, iron(II), using a UV-2501PC spectrophotometer (Shimadzu, Kyoto, Japan). Sulfide was analyzed using the Cline method^3^ and iron(II) and iron (III) using the ferrozine assay ^4^. Major anions and cations were detected and quantified by ion chromatography: An IonPac® CS12A-5µm cation-exchange column (Thermo Fisher Scientific Inc., Waltham, Massachusetts, U.S.A.) and eluent 20 mM methanesulfonic acid were used for major cations, whereas an IonPac® AS18-4µm anion-exchange column (Thermo Fisher Scientific Inc., Waltham, Massachusetts, U.S.A.) with a gradient eluent KOH (from 0.0 to 30 mM) was used for major anions. Trace metals Fe, Co, Cu, Sr, Ni, Mn, Cr, Zn and Al were measured using inductively coupled plasma mass spectrometry (ICP-MS), Agilent 8900 Triple Quadrupole (Agilent Technologies Inc., Santa Clara, California, U.S.A.), whereas all samples for trace elemental analysis were prepared in dilutions with 0.1 M HNO_3_ (final concentration, ultra-pure grade, MilliporeSigma, Burlington, Massachusetts, U.S.A.)

The sampled porewater was filtered on site for DNA analysis, using a sterile filtration device equipped with 0.2 μm polycarbonate membranes (MilliporeSigma, Burlington, Massachusetts, U.S.A.). For the first timepoint (removal in September 2014) the following method was used ^1^:

“Four filtrations of 250 mL each were performed. DNA was extracted from filtered water samples using a slightly modified protocol from the DNA Spin kit for Soil (MP Biomedicals LLC, Irvine, California, U.S.A.) and then purified using the Genomic DNA Clean & Concentrator purification kit (Zymo Research Corp., Irvine, California, U.S.A.). The total amount of DNA extracted was 19.5 ng and the final suspension had a concentration of 0.195 ng/μL.” (Smart *et al.*  ^2^)

Subsequent porewater samples were processed similarly but with the following differences: Three filtrations of 200 mL each were performed and the DNA was extracted using a phenol/chloroform approach as reported by Bagnoud *et al*. ^5^. However, the polycarbonate filter was not removed prior to the phenol treatment, because polycarbonate is soluble in phenol, thus a potentially higher DNA yield could be achieved. All used reagents for the DNA extraction are of molecular biology grade and care was taken to not introduce any contaminant DNA. Low amounts of recovered DNA can be attributed to two effects: a) the low biomass as starting point and b) the presence of Opalinus Clay particles that scavenge DNA.

## DNA amplification from borehole porewater

Borehole porewater was sampled for chemical analyses, (**Table S5**) and DNA microbiome profiling, (**Table S6**). 16S rRNA V4 region gene amplicons were generated used the PCR reaction mix KOD Hot Start (Merck KGaA, Darmstadt, Germany) (**1**) which contains the following: a 1x concentration of a proprietary Buffer for KOD Hot Start DNA polymerase, 1.5 mM MgSO_4_, 0.2 mM of each dNTP’s, 0.3 µM of each 515F-MiSeq and Pro806bR-MiSeq primers and 0.02 U/µL KOD Hot Start polymerase and 10µL of a DNA template to amplify the V4 region of the 16S rRNA gene from DNA extracted from the borehole porewater. The PCR was carried out for a total of 35 cycles (**2**). Below, the primer sequences are shown in bold along with the overhang sequence for Illumina MiSeq (not in bold):

515F-MiSeq, Parada *et al.*^13^

5’-TCG TCG GCA GCG TCA GAT GTG TAT AAG AGA CAG **GTG YCA GCM GCC GCG GTA A**-3’

Pro806bR-MiSeq, Appril *et al.*^14^

5'-GTC TCG TGG GCT CGG AGA TGT GTA TAA GAG ACA G**GG ACT ACN VGG GTW TCT AAT**-3'

The obtained PCR products were sent to the commercial sequencing laboratory Research and Testing Laboratory (RTL Genomics, Lubbock, Texas, U.S.A.) for sequencing on an Illumina MiSeq platform with a 2 x 250 bp paired-end read configuration to obtain a approx. 200 bp overlap. Raw sequencing reads were preprocessed (merging, quality filtering) with USEARCH^15^ version 11, allowing a maximum of 10 bases of differences between the paired-end reads. Operational taxonomic units (OTUs) were assigned with UPARSE^16^, and chimeras were removed utilizing UNOISE2^17^. Taxonomies of OTUs were assigned using SINTAX^23^, based on the published and curated 16S RDP database version 16, training set (2016)^24^, release 11. The number of high-quality reads was rarefied to 2,500 and imported to ampvis2^25^ which was used to analyze the dataset for similarities and explanatory variables.

**Table S1:** Composition of PCR solution used to amplify the V4 region of the 16S rRNA gene from DNA extracted from the borehole porewater.

|  | 50 µL reaction = 40 µL mastermix + 10 µL DNA | |
| --- | --- | --- |
|  | µL per sample | Final concentration |
| DEPC H_2_O | 20 | - |
| 10 x Buffer | 5 | 1 x |
| 2 mM dNTP mix | 5 | 0.2 mM |
| 25 mM MgSO_4_ | 3 | 1.5 mM |
| 5 µM forward primer | 3 | 0.3 µM |
| 5 µM reverse primer | 3 | 0.3 µM |
| KOD Hot Start polymerase | 1 | 0.02 U/µL |
| Total volume | 40 |  |

**Table S2:** Temperature cycles of PCR program used to amplify the V4 region of the 16S rRNA gene from DNA extracted from the borehole porewater.

| Temperature ºC | Duration |  |
| --- | --- | --- |
| 95 | 2 min |
| 95 | 25 sec | x34 |
| 55 | 15 sec |
| 70 | 15 sec |
| 70 | 3 min |  |

## Bentonite sampling and microbial cultivation analyses

The retrieved bentonite core was cut and sampled with disinfected knives and spatulas (70% ethanol) within an argon gas filled glovebox and packed in sterile sampling bags, then packed in two layers of Mylar™ bags with an argon gas atmosphere and kept at 4°C. In order to observe any variation in the distribution of microbial activity within the bentonite and around the corrosion coupons, samples were taken at various locations (interior and exterior parts of the bentonite core). The samples were transferred to the laboratories of EPFL, Lausanne, Switzerland, and stored at 4˚ C until analysis. The samples were cut into smaller pieces, using disinfected knives within a disinfected, nitrogen-filled glovebox, with the aim to remove potentially contaminated areas at the surface of the bentonite core. Using DNA-free forensic swabs (Sarstedt AG & Co KG, Nümbrecht, Germany), point-of-interest (POI) samples were obtained from various locations on the sintered stainless-steel filter, on the bentonite core surface (e.g., observed black spots) but also from bentonite adjacent to the embedded coupons. Swab samples were obtained systematically from surfaces such that the plain filter interior and the plain bentonite core surface, carefully excluding the aforementioned points-of-interest. The swab samples were kept dry and stored at -20˚ Celsius and transferred to EPFL and maintained frozen until analysis.

Enumeration of microorganisms followed the procedure based on the work by the Atomic Energy of Canada Limited (AECL) / Énergie atomique du Canada limitée (EACL) for the 'Analysis of rock, soil and clay for microbes' by Hamon ^6^:

A representative sample of bentonite was weight (wet weight) and chopped into small pieces which were placed in a volume of sterile, anoxic phosphate-buffered saline solution (1xPBS, volume depended on mass of bentonite such that a ten-fold dilution per mass is reached) to release microbial cells by shaking at 300 rpm for 60 minutes on a horizontal shaker plate within a sealed, anoxic, sterile serum bottle (mounted horizontally). A sample for cultivation was obtained from the middle of the suspension after one minute of sedimentation. The obtained suspension was then diluted in anoxic, sterile 1xPBS in 10-fold dilution steps up to a final dilution of 10^-6^ of the initial weight and used as an inoculum for the enumeration of microbial cells. Each dilution was plated in triplicates once for the enumeration of aerobic heterotrophic microbes and for anaerobic heterotrophic microbes using the following procedure: 1 ml of the dilution was pipetted into a sterile culture dish and 12-15 mL semi-solid R2A medium (Reasoner&Geldreich ^7^), cooled down to 45-50˚C, was added. The composition of the R2A medium is summarized in **Table S3**. The liquids were mixed by carefully swirling in all possible directions without spilling. After about 10 minutes, the R2A medium was solid and the triplicate plates were grouped together, inverted and the plates designated for the enumeration of anaerobic microbes transferred into an anaerobic jar to be transported to a heated glovebox (30˚C) and were incubated for three weeks. Plates designated for aerobic microbes were transferred to an incubation room (30˚C) and allowed to grow for three days. Enumeration of sulfate-reducing bacteria (SRB) was performed with the most probable number (MPN) method, as described by Hamon ^8^ . Hungate tubes were filled with 9 mL of sterile, anoxic Postgate’s Medium B as described by DSMZ ^9^ ,**Table S4** , and serial dilutions from 10^-1^ to 10^-5^ were made (in triplicates), using the same inoculum as for heterotrophic anaerobes and aerobes. The tubes were sealed to maintain anoxic conditions during seven weeks of incubation at 30˚C. The MPN method used is described by Hamon *et al.*  ^8^ and the MPN table used is published by the U.S. Food and Drug Administration in the Bacteriological Analytical Manual ^10^.

Figure S4: Bentonite core sampling: a) Slicing of a retrieved core into 6 slices; slices 1 to 5 are used for microbial investigations whereas the sixth slice is used for the determination of the bentonite dry density. b) Subsampling of the bentonite half-cylinder slices within a disinfected glovebox: (from left to right) a quarter is left as a backup, the other one is cut with sterile scalpel blades to remove potentially contaminated areas. The remaining slice was cut into 4 subsamples, representing an interior and an exterior part of the bentonite sample, for each a sample for cultivation (enumeration of microbes) and a subsample for DNA extraction.

Table S3: R2A medium for anaerobic and aerobic heterotrophs^7^. A total volume of 3.6 liters was aliquoted in 500 mL bottles, pH=7.2±0.1, and the anoxic medium flushed with sterile filtered nitrogen for 20 min.

| **Mass** [g] | **Substance** |
| --- | --- |
| 1.80 | Yeast-Extract |
| 1.80 | Bacto-peptone |
| 1.80 | Vitamin Assay Casamino Acids |
| 1.80 | Glucose (D-glucose) |
| 1.80 | Soluble starch |
| 1.08 | K_2_HPO_4_ |
| 1.08 | MgSO_4_ $\cdot$ 7H_2_O |
| 0.18 | Na-pyruvate |
| 6.00 per 500 mL aliquot | Agar |

Table S4: Postgate's Medium B for sulfate-reducing bacteria, composition according to DSMZ^9^. Total volume of 2 liters, pH=7-7.5

| **Mass** [g] | **Substance** |
| --- | --- |
| 7.0 | Na-lactate |
| 4.0 | MgSO_4_ $\cdot$ 7H_2_O |
| 2.0 | NH_4_Cl |
| 2.0 | CaSO_4_ |
| 2.0 | Yeast Extract |
| 1.0 | KH_2_PO4 |
| 1.0 | FeSO_4_ $\cdot$ 7H_2_O |
| 0.2 | Ascorbic acid |
| 0.2 (add as 20 mL solution) | Thioglycolic acid |
| 0.0008 (or 7mL of 0.025% solution) | Resazurin |

## DNA extraction from bentonite modules and swab samples

The extraction of DNA from bentonite was performed according to the recommendations by Engel *et al*.^11,12^ Representative samples of the interior and exterior of the bentonite core, adjacent to the samples obtained for the enumeration were used for DNA extraction using the DNeasy® PowerMax® Soil Kit (QIAGEN AG, Hilden, Germany) with a slightly modified protocol: samples of 2 ± 0.1 grams of bentonite were vortexed for 1 minute in 15ml PowerBead® solution, containing beads. 1.2 mL of Solution C1 was added and the samples were again vortexed for 30 seconds. After 30 minutes incubation at 65˚C, the samples were homogenized for 10 minutes at 30 Hz using a Mixer Mill MM 400 (Retsch GmbH, Haan, Germany). The following steps of the DNA extraction were carried out as suggested by QIAGEN. The extracted DNA was eluted in 2.1 mL of solution C6. The obtained DNA was further purified and concentrated using an isopropanol-ethanol precipitation method: 4µL/mL co-precipitant linear polyacrylamide GenElute® (molecular biology grade, MilliporeSigma, Burlington, Massachusetts, U.S.A.) was added to the eluted DNA, along with 0.1 volumes of 5 M NaCl (molecular biology grade, Merck KGaA, Darmstadt, Germany) and 1 volume of isopropanol (molecular biology grade, MilliporeSigma, Burlington, Massachusetts, U.S.A.) and gently mixed, left overnight at -20˚C to incubate. The precipitation was performed by 30 minutes centrifugation at 13’000 g at 4˚C using an Avanti® J-26 XP centrifuge (Beckman Coulter Inc., Brea, California, U.S.A.). The obtained DNA-polyacrylamide pellet was washed with sterile, -20˚C cold ethanol, 80 vol.%. This ethanol solution was prepared by mixing autoclaved MilliQ water (80-minutes, 121 °C) and molecular biology grade ethanol (Merck KGaA, Darmstadt, Germany). The DNA and ethanol solution was centrifuged for another 30 minutes at 13’000 g at 4˚C. Within a sterile laminar flow hood, the washed DNA pellet was air-dried for 10-20 minutes before resuspension in 125 µL of elution buffer (solution C6). After one-hour incubation at 4˚C, the eluted DNA was briefly centrifuged and transferred to 2 mL Soreson™ Dolphin tubes for storage at -20˚C until DNA analysis.

DNA from coupon imprints onto the bentonite were extracted in the same way with the only difference being that only bentonite close to the metal coupons was collected by scraping off a sample using new, sterile scalpel blades for each sample, within a sterile laminar flow box, thus the amount of bentonite varied between 0.1 and 1.9 grams of bentonite.

DNA from forensic swabs (Sarstedt AG & Co KG, Nümbrecht, Germany) and bentonite cuts were also extracted following the recommendations by Engel *et al*.^11,12^: A slightly modified protocol of the DNeasy® PowerSoil® Kit (OIAGEN AG, Hilden, Germany) was used: Within a sterile laminar flow hood the tips of swabs were cut with flame-sterilized scissors into PowerBead® tubes and briefly vortexed. Cuts of bentonite point-of-interests were added similarly within the laminar flow hood and the mass was recorded, varying between 0.003 and 0.182 grams. After addition of solution C1, the samples were briefly vortexed and incubated for 10 minutes at 70˚C. Homogenization was carried out with a Precellys 24 homogenizer (Bertin Technologies SAS, Montigny-le-Bretonneux, France), for 45 seconds at 6’000 rpm. The following steps are carried out as indicated in the kit manual, except for the elution of the extracted DNA: 65 µL of elution buffer C6 were applied to the center of the DNA binding membrane of a MB Spin Column. After a centrifugation step, 1 min at 8’000 g, the DNA was collected in a 2mL Soreson™ Dolphin tube and stored at -20˚C until DNA analysis.

All DNA was quantified using the Qubit® ds-DNA HS Assay Kit (Thermo Fisher Scientific Inc., Waltham, U.S.A.) according to the manufacturer’s protocol. The fluorescence signal was measured with a Qubit® 2.0 Fluorometer (Thermo Fisher Scientific Inc., Waltham, U.S.A.).

## DNA amplification from bentonite and module samples

DNA from bentonite and swabs was amplified using quantitative PCR. We used a modified protocol based on the *Quick*-16S NGS Library Prep Kit (Zymo Research Corp., Irvine, California, U.S.A.). The utilization of the SYBR Green fluorescence signal during the PCR limits the generation of chimeric DNA amplicons, thus greatly improving the control over the amplification process. The provided kit allows the amplification of the V4 region of the 16S rRNA gene and allow the multiplexing of up to 376 samples into a single MiSeq lane. We decided to sequence on two MiSeq lanes (lane 1: 280 uniquely barcoded samples, lane 2: with 243 uniquely barcoded samples). Additionally, we took advantage of the fluorescence signal to a semi-quantitative assessment of the 16S rRNA gene copy number. It is semi-quantitative because of two major limitations: i) no amplification replicates instead of the usual triplicates needed and ii) employing a standard curve method, using a pGEM-T plasmid (Promega Corp. Madison, U.S.A.) containing a *E.* coli 16S rRNA gene copy as a standard. The latter is known to perform poorly for the quantification of environmental microbial communities^21^. Nevertheless, qPCR provided insight into the relative quantities of rRNA, and thus improved the robustness of the DNA sequencing interpretation. The 6-point standard curve was performed from 10^7^ copies per µL, down to 100 copies per µL in ten-fold dilution steps. Together with a positive control (Zymo Microbial Community Standard®) and a negative control, each 96-well plate of the *Quick*-16S NGS Library Prep Kit could be used to amplify 88 samples. The amplification was carried out on a LightCycler® 480 Instrument (F. Hoffmann-La Roche AG, Basel, Switzerland) using LightCycler® 480 Multiwell plats (96-well, white, F. Hoffmann-La Roche AG, Basel, Switzerland). For this system, the fluorescence threshold was determined at 24.63, which indicates the onset of an exponential increase of the fluorescence signal based on the amplification of a targeted DNA sequence. The amplification, barcoding and 16S rRNA-library generation of DNA samples from bentonite, modules swabs and contamination controls swabs were carried out as suggested from Zymo Research Corp. PCR efficiencies were calculated using LinRegPCR^22^, and the 16S rRNA gene copy number for the samples on each plate were calculated based on the standard curve method with the values obtained on the same plate^21^. The 16S rRNA-library was sequenced on an Illumina MiSeq (Illumina Inc., San Diego, U.S.A.) platform at the Lausanne Genomics Technologies Facility (University of Lausanne, Switzerland), applying 10 pM of the 16S-library with 15% Phi-X in a paired-end 300bp mode.

The processing of raw reads was carried out as recommended in the USEARCH v 11 manual: Raw sequencing reads were preprocessed (merging, quality filtering) with USEARCH^15^ version 11,allowing a maximum of 10 bases difference between the paired-end reads. Operational taxonomic units (OTUs) were assigned with UPARSE^16^, and chimeras were removed utilizing UNOISE2^17^. Taxonomies of OTUs were assigned using SINTAX^23^, based on the published and curated 16S RDP database version 16, training set (2016)^24^, release 11. The number of high-quality reads was rarefied to 2,500 and imported to ampvis2^25^ which was used to analyze the dataset for similarities and explanatory variables. Results are visualized using the R^26^-package ggplot2^27^ to create balloon plots representing major OTUs on the family level for each sample, where the balloon size is indication of the relative contribution within the respective sample (in %) and samples obtained from similar locations were averaged for each module.

## Water content, CFU & MPN per gram dry weight, observed dry density of bentonite

For each bentonite core, one slice was used to determine the water content and the dry density. A cheese knife was used to cut the bentonite slice into six approximately equal parts. The dimensions of the slices were measured with a ruler and the wet weight ($m_{sample wet}$) was rapidly recorded using an analytical scale before air-drying impacted the weight. The bentonite was dried in a laboratory oven at 105˚C. Dry weights of cooled samples (in moisture-free conditions) were recorded after 24 hours, 48 hours and after seven days. The lowest weights were recorded for all samples after seven days and the mass of water ($m_{water}$) was calculated on that basis. The moisture content relative to wet sample was calculated using

$$\%moisture relative to wet sample=\frac{m_{water}*100}{m_{sample wet}}$$

and the moisture content relative to dry sample:

$$\%moisture relative to dry sample=\frac{m_{water}*100}{m_{sample dry}}$$

$$water content relative to wet mass=\frac{m_{water}}{m_{sample wet}}$$

Water content relative to wet mass and wet mass were used to calculate the colony forming units (CFU) and most probable number (MPN) per gram of Wyoming Bentonite dry weight. The CFU calculation is shown as an example below:

$$\frac{CFU}{dry bentonite mass}=\frac{CFU}{wet mass* (1-relative water content)}$$

Additionally, the bentonite dry densities were obtained by

$$\rho_{dry}=\frac{m_{sample wet}-m_{water}}{V_{wet}}$$

## Oxygen desorption from Wyoming bentonite

Eight glass serum bottles (V=1220 mL) were filled with 300 grams of as-received MX-80 Wyoming bentonite and sealed with rubber stoppers. Five additional bottles were sealed without bentonite and used as controls for bottle and stopper tightness. The air trapped within the sealed bottles was removed during 30 min flushing with nitrogen at atmospheric pressure, the bottle containing bentonite were shaken every 5 minutes to ensure removal of air trapped between bentonite grains. The bottles were stored in the dark at room temperature. 10 mL of the gas phase (a total of approx. 1000 mL), were sampled at approx. 24h intervals for oxygen detection in a gas chromatography system. The removed gas volume was replaced by anoxic nitrogen. The gas chromatography system Varian GC-450 (Agilent Technologies, Santa Clara, CA, U.S.A.) used is equipped with a molecular sieve column (CP81071: 1.5m*1/8'' ultimetal molsieve 13 9 80-100 mesh) and the detection of oxygen was performed using a thermal conductivity detector (TCD). After the experiment, three bentonite samples were sieved for particles smaller than one millimeter, dried in an oven for two hours at 200 degrees Celsius and analyzed for their specific surface area (BET-analysis) using a TriStar II Plus system (Micromeritics Instrument Corporation, Norcross, Georgia, U.S.A.). The BET-analysis was carried out by Lionel Sofia of the service department within the Laboratory of Construction Material at EPFL, Lausanne, Switzerland.

# Results

## Borehole water composition and microbiome

Additional information about the borehole water chemical composition at four timepoints is presented in **Table S5**. Additionally, the microbial composition of the borehole water can be found in **Table S6**. While the amplified V4 region of the 16S rRNA gene allows the detection of both Archaea and Bacteria, only Bacteria were found amongst the major contributors to the microbial community in the porewater (and in the bentonite modules).

Table S5: Samples of filtered porewater were collected at four timepoints and the chemical composition was investigated. Dashes indicate no analysis.

| Component | **January 2013**^1^ | **September 2014** | **July 2017** | **July 2018** | Unit |
| --- | --- | --- | --- | --- | --- |
| **Fe(II)** | 1.3 | 76.3^2^ | 0.3 | 31.6 | μM |
| **H_2_S** | 7.4 | - | <0.1 | <0.1 | μM |
| **Co** | <10 | - | <50 | <500 | μg L^−1^ |
| **Cu** | 13 | - | <50 | <500 | μg L^−1^ |
| **Sr** | 60.657 | - | 18.387 | 26.467 | mg L^−1^ |
| **Ni** | <10 | - | 69.3 | <500 | μg L^−1^ |
| **Mn** | 53 | - | 125.9 | <500 | μg L^−1^ |
| **Cr** | 31 | - | <50 | <500 | μg L^−1^ |
| **Zn** | <10 | - | <50 | <500 | μg L^−1^ |
| **Al** | <10 | - | <50 | <500 | μg L^−1^ |
| **Na^+^** | 313.6 | 267.1 | 257.9 | 258.7 | mM |
| **NH^+^_4_** | 0.3 | - | 4.6 | 0.9 | mM |
| **Mg^2+^** | 15.0 | 3.0 | 13.0 | 9.8 | mM |
| **K^+^** | 1.9 | 9.00 | 3.8 | 3.4 | mM |
| **Ca^2+^** | 18.8 | 15.3 | 12.3 | 13.4 | mM |
| **F^-^** | 0.1 | - | 0.03 | <0. 5 | mM |
| **Cl^-^** | 290.3 | 226.5 | 264.6 | 274.8 | mM |
| **NO^-^_2_** | <0.005 | - | <0.05 | <0.5 | mM |
| **SO_4_^2–^** | 15.6 | 23.3 | 21.8 | 22.5 | mM |
| **Br^-^** | 0.5 | - | <0.05 | <0.5 | mM |
| **NO_3_ −** | <0.005 | - | 0.16 | 0.09 | mM |
| **PO_4_ ^3−^** | <0.005 | - | 0.04 | <0.5 | mM |
| **pH** | 8.2 | - | 8.30 | 8.45 |  |
| **EH** | -88 | -88 | -271.8 | -230.1 | mV |

Table S6: Operational taxonomical units of the porewater microbial community. Contributions larger than 1% to the community for at least one timepoint are listed. A detailed investigation of the initial timepoint, January 2013, was reported in Bagnoud et al.^1^. The contribution of sulfate-reducing bacteria decreases from the first to the second timepoint, but increases again from 2017 onwards. Generally, the 2017 and 2018 samples exhibit a more diverse microbial community in the porewater than earlier timepoints.

| Taxonomy (genus or family) | **01/2013*^1^* Rel. %** | **09/2014 Rel. %** | **07/2017 Rel. %** | **07/2018 Rel. %** |
| --- | --- | --- | --- | --- |
| *Pseudomonas* | 25.9 | 87.3 | 30.9 | 24.9 |
| *Desulfotomaculum* | 58.9 | 2.4 |  |  |
| *Desulfosporosinus* | 7.4 | 1.7 | 9.3 | 2.0 |
| *Natronincola anaerovirgula* | 2.3 |  | 1.7 | 2.0 |
| Peptococcaceae | 1.0 |  | 2.5 | 22.1 |
| *Sporotomaculum* |  |  | 4.8 | 4.4 |
| *Hydrogenophaga* |  |  | 15.3 | 5.0 |
| *Desulfitobacter* |  |  | 1.6 | 5.5 |
| *Desulfococcus* |  |  | 1.0 | 2.5 |
| Desulfobulbaceae |  |  | 3.4 | 5.9 |
| Acidobacteria iii1-8 DS-18 |  |  | 1.0 | 1.5 |
| *Thiobacillus* |  |  | 2.5 |  |
| *Desulfurispora* |  |  | 1.3 |  |
| *Dethiobacter* |  |  |  | 1.1 |
| *Devosia* |  |  |  | 1.0 |
| *Gracilibacter* |  | 2.2 |  |  |
| *Poseidonocella* |  | 1.3 |  |  |
| Natranaerobiales | 2.2 |  |  |  |
| Beijerinckiaceae |  |  | 1.5 |  |
| Rhodobacteraceae |  |  |  | 1.3 |
| Clostridia |  |  | 3.8 | 6.8 |
| Bacteria |  |  | 6.9 |  |

## Bentonite properties, cultivation of (an)-aerobic heterotrophs and sulfate-reducing bacteria and 16S rRNA gene quantification


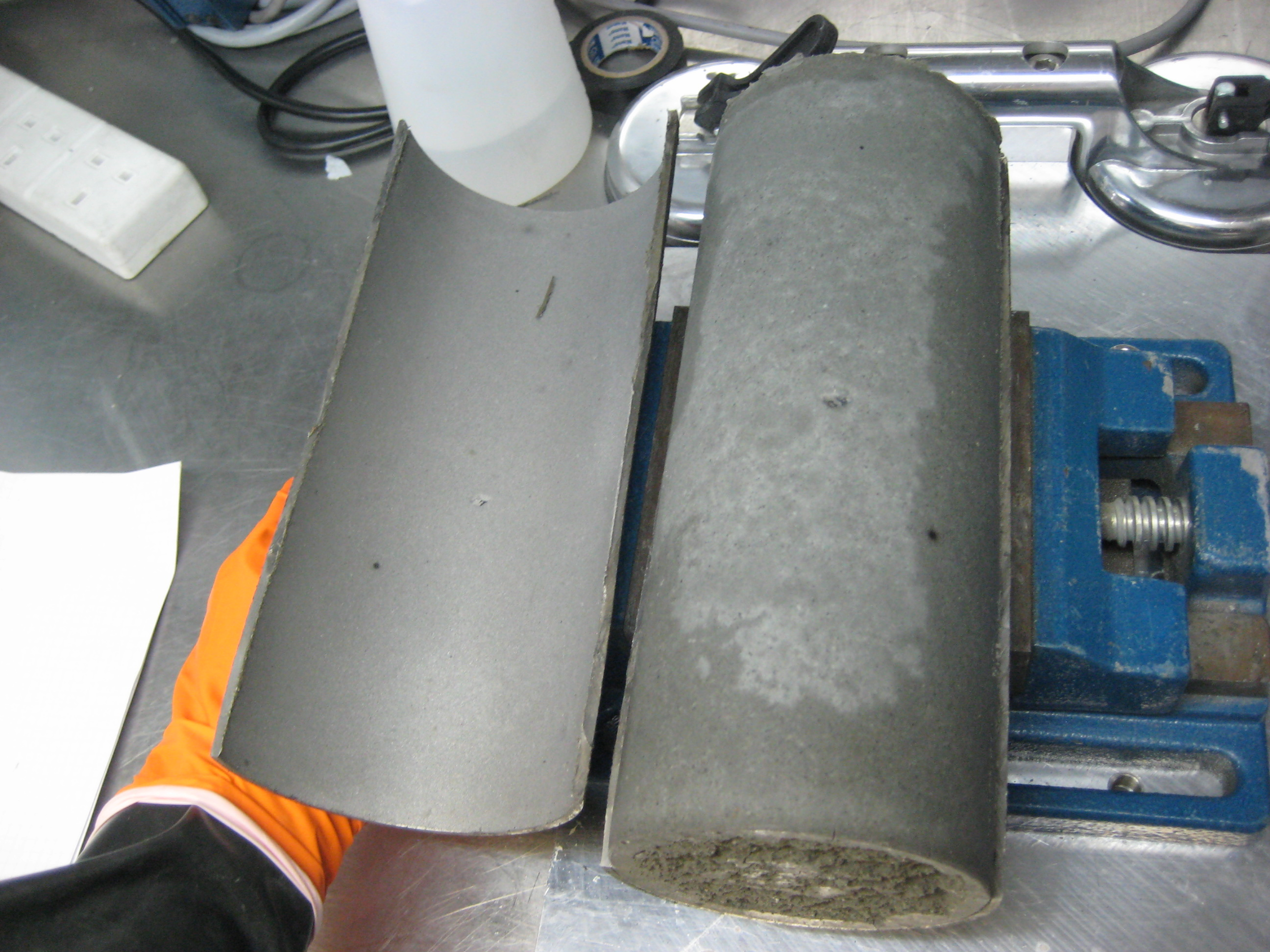


**Sintered filter cut in half to reveal the bentonite cylinder**

**Bentonite cylinder**

Figure S5: Photograph of bentonite core (M13, deployed for 2.5-years, 1.45 g/cm^3^ powder/pellets): the surface features include localized black spots which are also observed on the sintered stainless-steel filter that was in contact with the bentonite. Arrows show black spots on the surface of the bentonite cylinder and their corresponding location on the sintered filter.


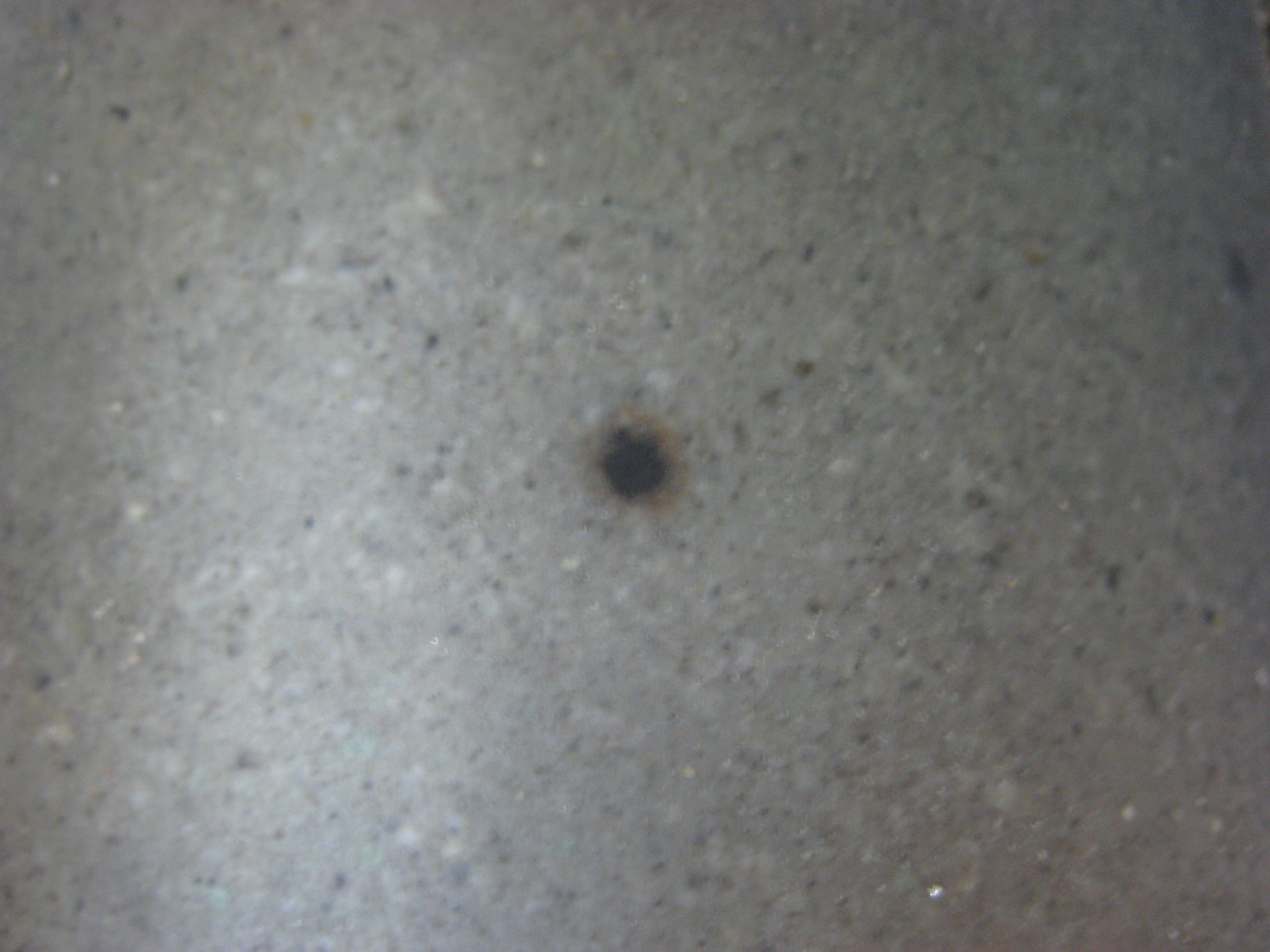

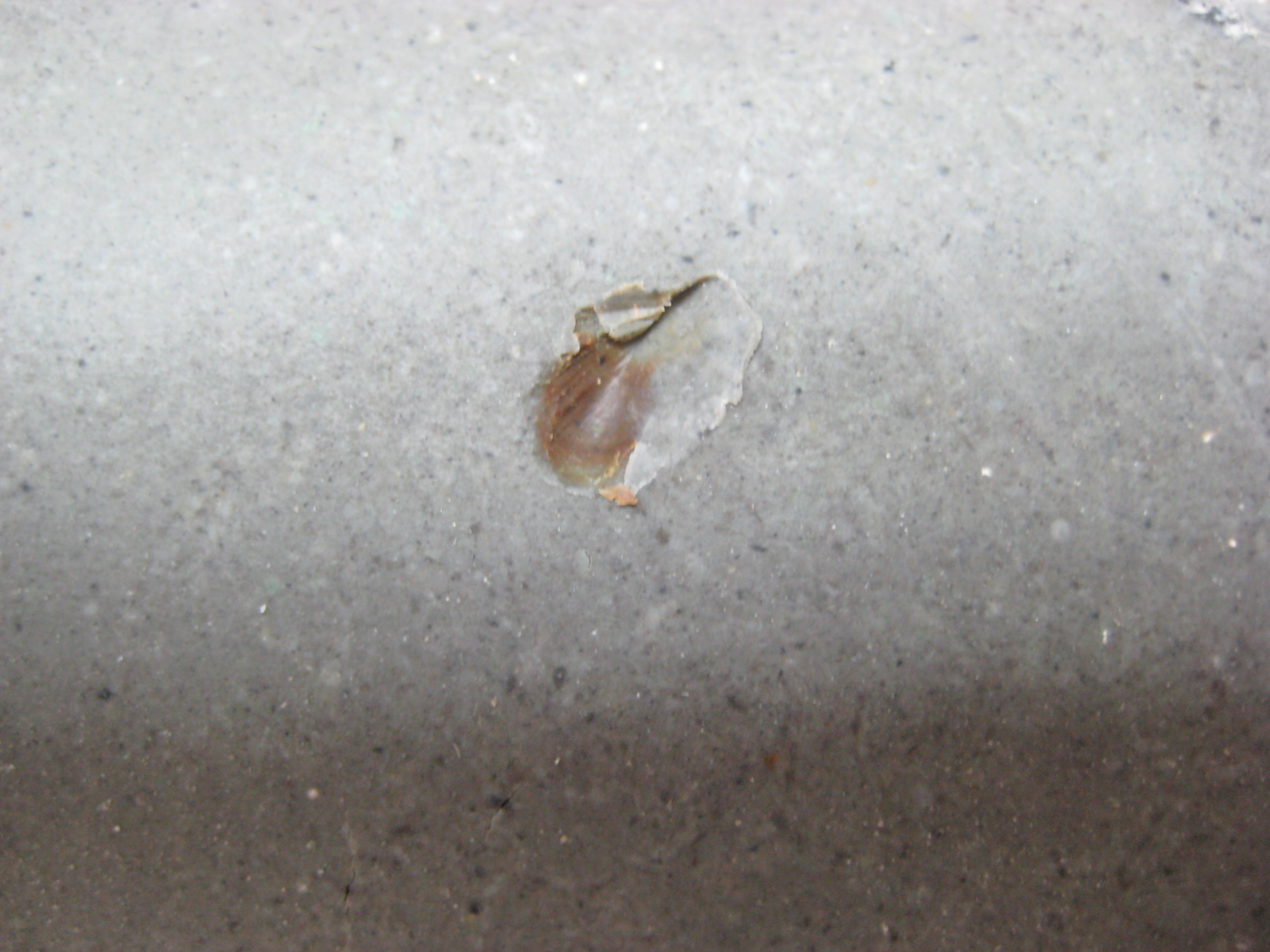


Figure S6: Black spots (top) observed on the bentonite core surface are potentially containing iron-sulfide minerals. Below the black spot, in approximately 2 mm depth we observed a brown color in the bentonite, suggesting iron oxide formation. Iron(II) may leach out of the sintered stainless-steel filter and react with sulfide within the porewater (black precipitates) or with bentonite-trapped oxygen, forming iron-oxides (brown precipitates).


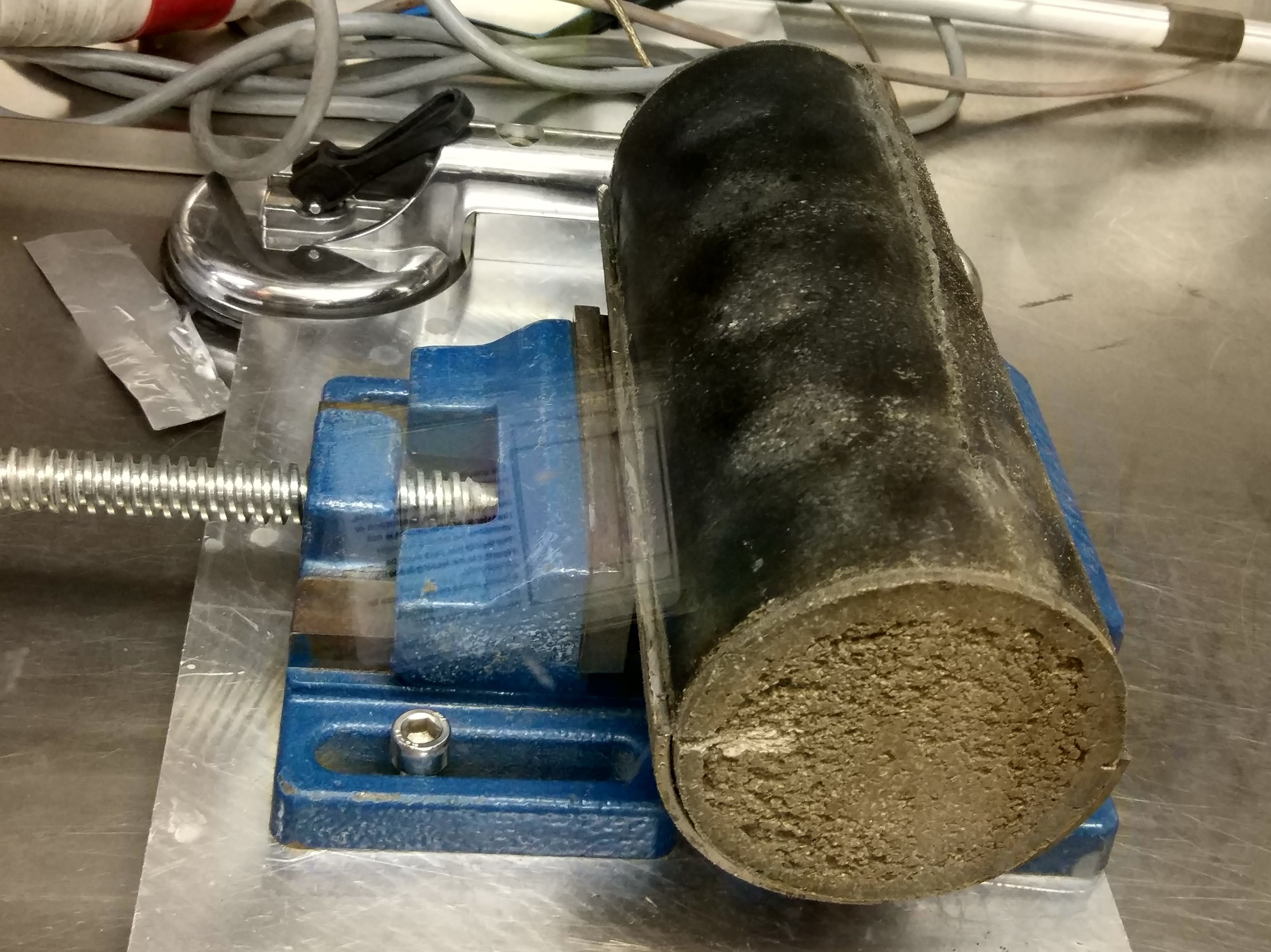


Figure S7: Bentonite core (M6, deployed for 5.5-years, 1.55 g/cm^3^) after removal of the top half of the sintered filter (the lower half is used as support during sampling). We observe a dark color on the surface, except for the places where the metal test coupons are placed within the bentonite, suggesting the potential interaction of metal coupons with sulfide-rich borehole water at the bentonite-borehole water interface. Corrosion data are not available for this timepoint.


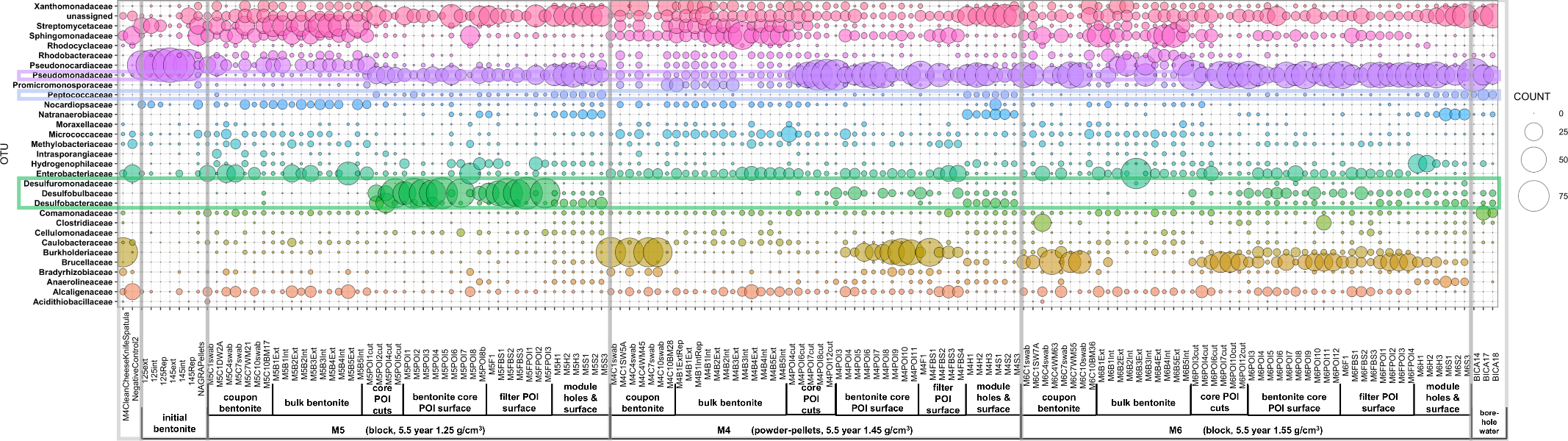


**Figure S8**: Balloon plot of three 5.5-year modules, M5 (block bentonite at 1.25 g/cm^3^), M4 (pellet-powder bentonite at 1.45 g/cm^3^), M6 (block bentonite at 1.55 g/cm^3^). Major OTUs at the family level (more than 1% rel. abundance) for individual samples from all sampled locations are plotted: POI=Point of interest. The samples are ordered from left to right: The first two are controls, followed by seven samples of the initial bentonite and then by module samples M5, M4 and M6 and finally three samples of the borehole water. For each module, samples are grouped from the interior towards the outside, starting with bentonite close to coupons, the bulk bentonite followed by the filter-bentonite interface, i.e. the bentonite core surface and the filter surface. Lastly, the samples related to the module holes and module surface are presented. In the highest dry-density module M6 (1.55 g/cm^3^), the bentonite in contact with the coupon exhibits an OTU (Brucellaceae (orange)) present at the bentonite core surface and the module holes and surface but not in bulk bentonite. This effect was only observed in one module and thus needs confirmation from future longer timepoints. Five typical porewater OTUs are indicated with boxes, highlighting the absence of sulfate-reducing bacteria (three OTUs in green box) in coupon and bulk bentonite samples. The five typical borehole porewater OTUs were observed on the bentonite core surface, in POI samples from the bentonite core and the filter surfaces, as well as the module holes and surface.


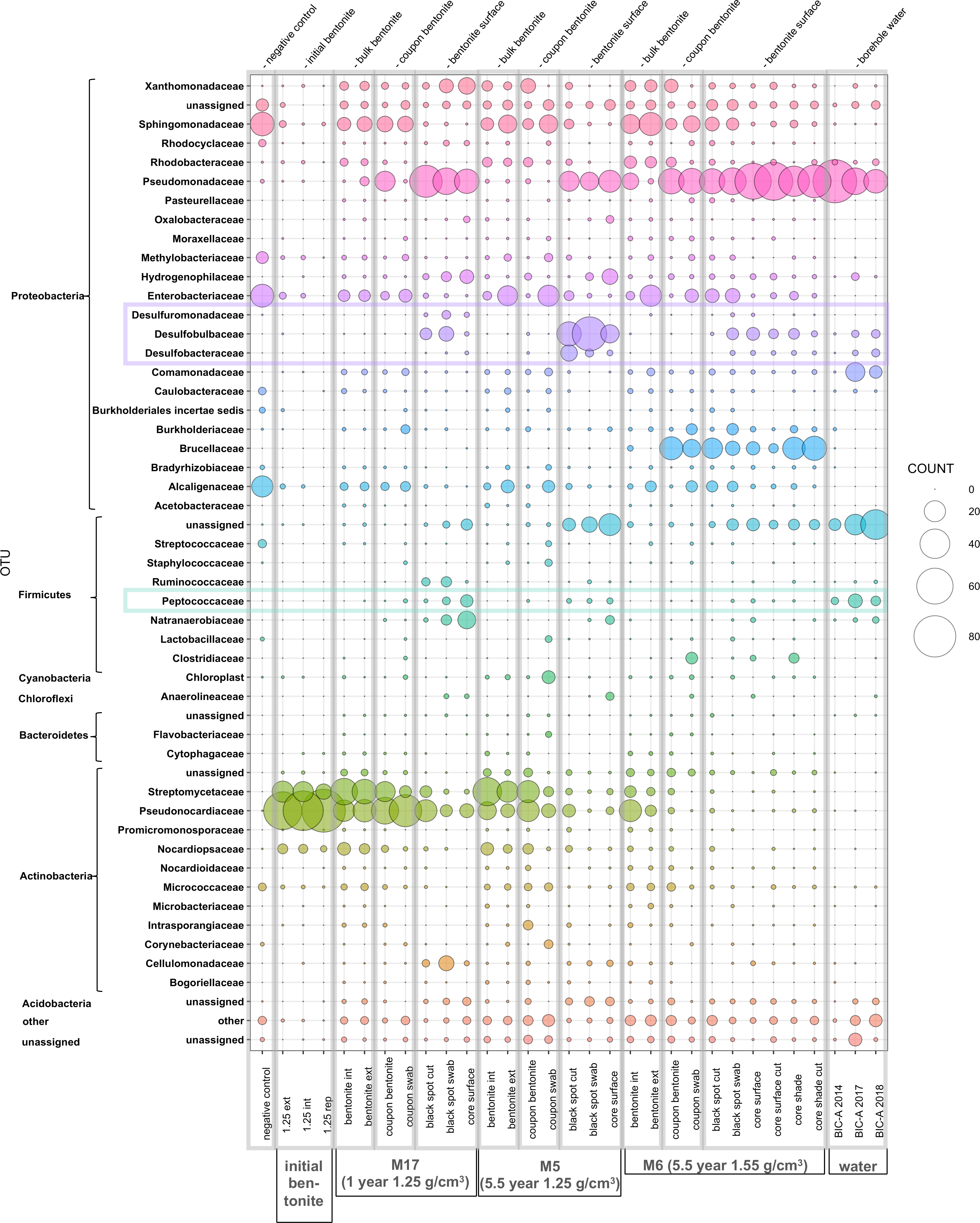


**Figure S9**: 16S rRNA gene V4 region amplicon profile of bentonite from three modules: M17 (1-year), M5 (5.5-years) both featuring low-density bentonite blocks (1.25 g/cm^3^), and M6 (5.5-years) consisting of a high-density bentonite block (1.55 g/cm^3^). The x-axis lists the samples grouped and averaged within their sample location group, while major contributions of operational taxonomic units (OTUs), at the phylum and family level, are listed on the y-axis. Each OTU is color-coded according to its Phylum and the size of the circle corresponds to its normalized (percent) occurrence within a given sample. Only OTUs with > 1% relative abundance within a sample and including three observations (above threshold) are shown; the remaining minor OTUs are represented within ‘other’. Shown are averages of sample groups: Control sample (negative control) (n=1); undeployed bentonite blocks (initial bentonite) (n=3); for each module (M17, M5 and M6), bentonite bulk interior (bentonite int) and exterior (bentonite ext) (n=5); bentonite adjacent to coupons (coupon bentonite) (n=4); swabs of bentonite in contact with coupons (coupon swab) (n=4), cuts/extractions of black spots on the bentonite cylinder surface (black spot cut) (M17 n=3, M5 n=4, M6 n=3); swabs of black spots (black spot swab) (M17 n=4, M5 n=7, M6 n=5); swabs of bentonite cylinder surface without visible features (core surface) (M17 n=1, M5 n=1, M6 n=1); cut/extract of bentonite cylinder surface (core surface cut) (M6 n=1); swab of discolored area on bentonite cylinder surface (core shade) (M6, n=4); cut/extract of discolored area on bentonite cylinder surface (core shade cut) (M6, n=2), borehole water community (n=3). The porewater samples from BIC-A are labeled according to the year of their sampling.

Additional information about the bentonite dry density, moisture content, and water activity is shown in **Table S7**.

Table S7: Moisture content calculated from wet and dry weights. Water activity data are only available for the first set of modules (1-3). ^A^Water weight x 100/weight of original sample. ^B^Water weight x 100/weight of dried sample. Table modified from Smart et al.^2^ with added moisture values for the second, third and fourth set of test modules.

| Module ID | Deployment time [years] | Target dry bentonite density before emplacement [g cm^-3^] | Moisture [%] wet weight^A^ | Moisture [%] dry weight^B^ | Water activity *a*_w_ |
| --- | --- | --- | --- | --- | --- |
| **1** | 1.5 | 1.45 | 24.3 | 32.1 | 0.956 (±0.0104) |
| **2** | 1.5 | 1.25 | 28.9 | 40.6 | 0.984 (±0.027) |
| **3** | 1.5 | 1.55 | 22.6 | 29.3 | 0.950 (±0.014) |
| **4** | 5.5 | 1.45 | 22.3 | 29.7 | - |
| **5** | 5.5 | 1.25 | 28.0 | 38.9 | - |
| **6** | 5.5 | 1.55 | 23.0 | 29.8 | - |
| **13** | 2.5 | 1.45 | 27.2 | 37.4 | - |
| **14** | 2.5 | 1.55 | 24.0 | 31.6 | - |
| **15** | 2.5 | 1.45 | 26.5 | 36.2 | - |
| **16** | 1.0 | 1.45 | 23.8 | 31.5 | - |
| **17** | 1.0 | 1.25 | 29.9 | 40.9 | - |
| **18** | 1.0 | 1.45 | 24.6 | 32.6 | - |

## 16S rRNA gene amplicon sequence analysis

The paired-end Illumina MiSeq run resulted in the following number of raw reads (before any quality filtering): Forward sequencing, per sample, average of 21’790 reads, median of 17’891 reads, minimum reads of 29, maximum reads of 264052. Reverse sequencing, per sample, average of 21’804 reads, median of 18’104 reads, minimum of 29 reads, maximum of 26’4052.

The merging process (performed with USEARCH) resulted in 8’302’101 pairs (8.3M) of which 7’683’715 were merged (7.7M, 92.55%). Between forward and reverse sequencing 4’697’746 alignments were observed with zero differences between their bases (56.59%), while 583’047 had too many different bases (> 10) (7.02%). 11’713 forward tails with Q <= 2 were trimmed (0.14%) and 25’509 reverse tails with Q <= 2 were trimmed (0.31%). 817 forward sequence reads were too short (< 64) after tail trimming (0.01%) and 3’665 reverse sequence reads were too short (< 64) after tail trimming (0.04%). For 30’857 reads no alignment found (0.37%), 0 read alignments were too short (< 16) (0.00%). In total, 8’059’428 staggered pairs (97.08%) were merged & trimmed, resulting in a mean alignment length of 289.54 bp and a mean merged length of 291.68 bp. The mean forward read expected error was determined at 0.35 while the mean reverse read expected error is at 1.22. The mean error of merged reads is expected at 0.05.

More information about sequencing depth (and alpha diversity) is given in section 4. APPENDI of this Supplementary.

## Oxygen desorption from Wyoming bentonite

As presented in Results & Discussion, the persistence of aerobic heterotrophs and the observation of a rather high relative contribution of OTUs belonging to aerobic families led us to hypothesize the longer-term presence of bioavailable oxygen within a nominally anoxic environment. In order to test this hypothesis, we performed a simple gas desorption test and could detect low levels of oxygen starting between 3 to 9 days in the initially oxygen-free gas phase of isolated bentonite (**Figure S10**)

oxygen (%)


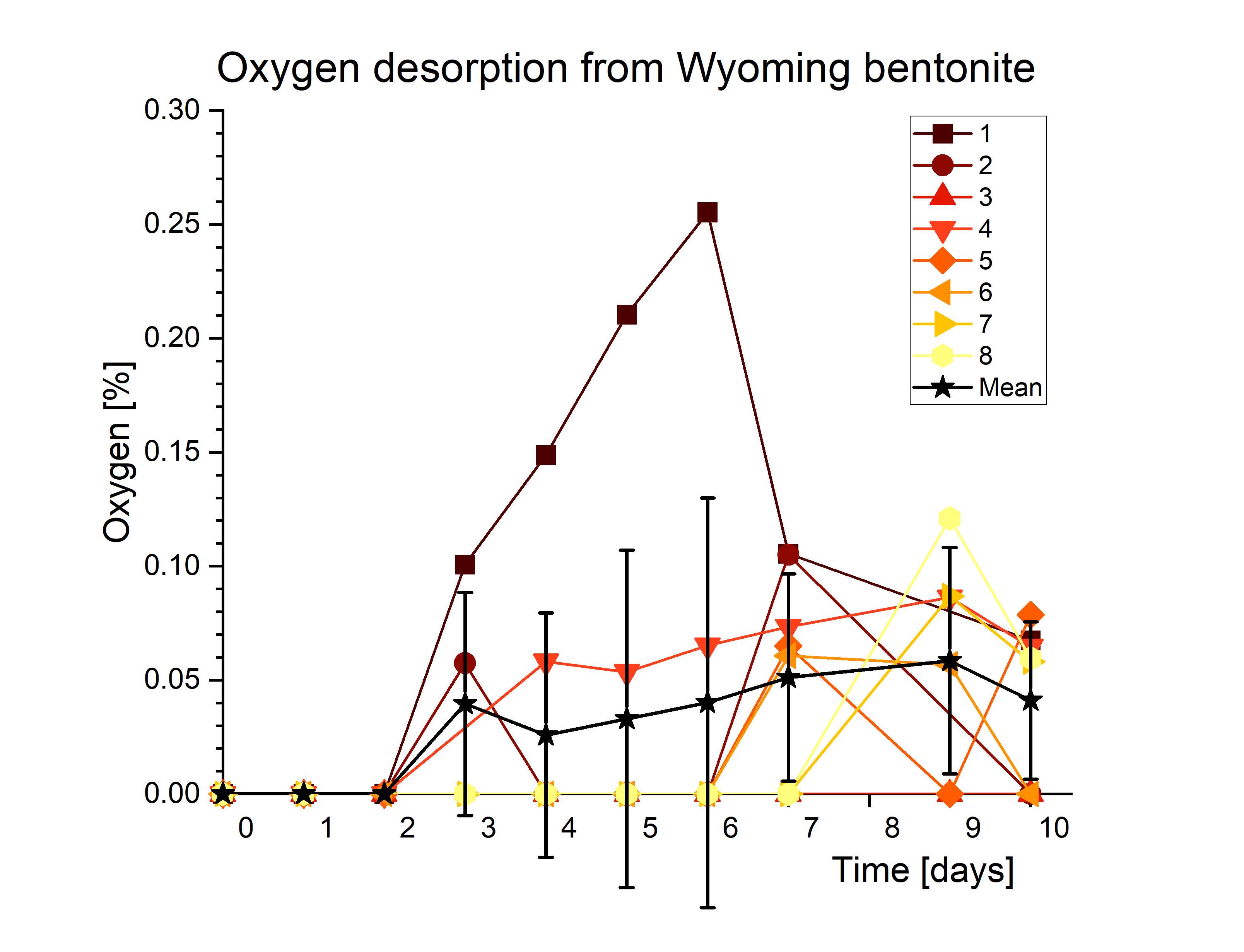


Figure S10: Oxygen quantification from bentonite gas phases, desorption within 10 days upon sealing within a nitrogen atmosphere. The results indicate desorption of oxygen from bentonite starting at earliest after three days (dark brown) but we observe that between 7 and 9 days (orange to yellow) are necessary to be able to detect approx. 0.05% oxygen (limit-of-detection).

Table S8 - Bentonite samples used for BET analysis to quantify the specific surface area of the used oxygen-desorption bentonite.

|  | Wyoming MX-80 sample **1** | Wyoming MX-80 sample **2** | Wyoming MX-80 sample **3** |
| --- | --- | --- | --- |
| **Mass** (as received, humid) [g] | 300 | 300 | 300 |
| **Water content** [%] | 0.45 | 0.43 | 0.52 |
| **BET specific surface** [m^2^/g] | 32.2909 | 27.9269 | 33.2951 |

# Download link for NAGRA report and figure data

The Nagra Research, Development and Demonstration (RD&D) Plan for the Disposal of Radioactive Waste in Switzerland - Technischer Bericht 09-06. (2009).

<https://www.nagra.ch/data/documents/database/dokumente/$default/Default%20Folder/Publikationen/NTBs%202001-2010/e_ntb09-06.pdf>

Supplementary figures S8-S10 were created with the data stored in the archive ‘Burzan_2021_Frontiers_Zenodo-opensource_supplementary.zip’ that can be downloaded from Zenodo (open-access, DOI): 10.5281/zenodo.4883637

**References**

(1) Bagnoud, A.; de Bruijn, I.; Andersson, A. F.; Diomidis, N.; Leupin, O. X.; Schwyn, B.; Bernier-Latmani, R. A Minimalistic Microbial Food Web in an Excavated Deep Subsurface Clay Rock. *FEMS Microbiol Ecol* **2016**, *92* (1). https://doi.org/10.1093/femsec/fiv138.

(2) Smart, N. R.; Reddy, B.; Rance, A. P.; Nixon, D. J.; Frutschi, M.; Bernier-Latmani, R.; Diomidis, N. The Anaerobic Corrosion of Carbon Steel in Compacted Bentonite Exposed to Natural Opalinus Clay Porewater Containing Native Microbial Populations. *Corrosion Engineering, Science and Technology* **2017**, *52* (sup1), 101–112. https://doi.org/10.1080/1478422X.2017.1315233.

(3) Cline, J. D. Spectrophotometric Determination of Hydrogen Sulfide in Natural Waters1. *Limnology and Oceanography* **1969**, *14* (3), 454–458. https://doi.org/10.4319/lo.1969.14.3.0454.

(4) Stookey, L. L. Ferrozine---a New Spectrophotometric Reagent for Iron. *Anal. Chem.* **1970**, *42* (7), 779–781. https://doi.org/10.1021/ac60289a016.

(5) Bagnoud, A.; Chourey, K.; Hettich, R. L.; de Bruijn, I.; Andersson, A. F.; Leupin, O. X.; Schwyn, B.; Bernier-Latmani, R. Reconstructing a Hydrogen-Driven Microbial Metabolic Network in Opalinus Clay Rock. *Nat Commun* **2016**, *7*. https://doi.org/10.1038/ncomms12770.

(6) Hamon, C. J. Analysis of Rock, Soil and Clay for Microbes - MIC.05 - AECL/EACL. AECL/EACL September 1, 2006.

(7) Reasoner, D. J.; Geldreich, E. E. A New Medium for the Enumeration and Subculture of Bacteria from Potable Water. *Applied and Environmental Microbiology* **1985**, *49* (1), 1–7.

(8) Hamon, C. J. MPN - Most Probable Number Method of Counting Viable Bacteria - MIC.02 - AECL/EACL. AECL/EACL January 5, 2003.

(9) DSMZ, G. C. of M. and C. C. Medium 63 - Postgate’s Medium. August 8, 2018.

(10) Nutrition, C. for F. S. and A. BAM Appendix 2: Most Probable Number from Serial Dilutions. *FDA* **2019**.

(11) Engel, K.; Coyotzi, S.; Slater, G.; Neufeld, J. D. Development of Protocols for Sampling and Assessment of Bentonite and Environmental Samples Associated with in Situ Proof Tests of Engineered Barrier Systems - NWMO-TR-2018-04. NWMO - Nuclear Waste Managment Organisation January 1, 2018.

(12) Engel, K.; Coyotzi, S.; Vachon, M. A.; McKelvie, J. R.; Neufeld, J. D. Validating DNA Extraction Protocols for Bentonite Clay. *mSphere* **2019**, *4* (5). https://doi.org/10.1128/mSphere.00334-19.

(13) Parada, A. E.; Needham, D. M.; Fuhrman, J. A. Every Base Matters: Assessing Small Subunit RRNA Primers for Marine Microbiomes with Mock Communities, Time Series and Global Field Samples. *Environmental Microbiology* **2016**, *18* (5), 1403–1414. https://doi.org/10.1111/1462-2920.13023.

(14) Apprill, A.; McNally, S.; Parsons, R.; Weber, L. Minor Revision to V4 Region SSU RRNA 806R Gene Primer Greatly Increases Detection of SAR11 Bacterioplankton. *Aquatic Microbial Ecology* **2015**, *75* (2), 129–137. https://doi.org/10.3354/ame01753.

(15) Edgar, R. C. Search and Clustering Orders of Magnitude Faster than BLAST. *Bioinformatics* **2010**, *26* (19), 2460–2461. https://doi.org/10.1093/bioinformatics/btq461.

(16) Edgar, R. C. UPARSE: Highly Accurate OTU Sequences from Microbial Amplicon Reads. *Nature Methods* **2013**, *10* (10), 996–998. https://doi.org/10.1038/nmeth.2604.

(17) Edgar, R. C. UNOISE2: Improved Error-Correction for Illumina 16S and ITS Amplicon Sequencing. *bioRxiv* **2016**, 081257. https://doi.org/10.1101/081257.

(18) Cole, J. R.; Wang, Q.; Cardenas, E.; Fish, J.; Chai, B.; Farris, R. J.; Kulam-Syed-Mohideen, A. S.; McGarrell, D. M.; Marsh, T.; Garrity, G. M.; Tiedje, J. M. The Ribosomal Database Project: Improved Alignments and New Tools for RRNA Analysis. *Nucleic Acids Res* **2009**, *37* (Database issue), D141–D145. https://doi.org/10.1093/nar/gkn879.

(19) Caporaso, J. G.; Kuczynski, J.; Stombaugh, J.; Bittinger, K.; Bushman, F. D.; Costello, E. K.; Fierer, N.; Peña, A. G.; Goodrich, J. K.; Gordon, J. I.; Huttley, G. A.; Kelley, S. T.; Knights, D.; Koenig, J. E.; Ley, R. E.; Lozupone, C. A.; McDonald, D.; Muegge, B. D.; Pirrung, M.; Reeder, J.; Sevinsky, J. R.; Turnbaugh, P. J.; Walters, W. A.; Widmann, J.; Yatsunenko, T.; Zaneveld, J.; Knight, R. QIIME Allows Analysis of High-Throughput Community Sequencing Data. *Nat Meth* **2010**, *7* (5), 335–336. https://doi.org/10.1038/nmeth.f.303.

(20) Quast, C.; Pruesse, E.; Yilmaz, P.; Gerken, J.; Schweer, T.; Yarza, P.; Peplies, J.; Glöckner, F. O. The SILVA Ribosomal RNA Gene Database Project: Improved Data Processing and Web-Based Tools. *Nucleic Acids Res* **2013**, *41* (Database issue), D590–D596. https://doi.org/10.1093/nar/gks1219.

(21) Brankatschk, R.; Bodenhausen, N.; Zeyer, J.; Bürgmann, H. Simple Absolute Quantification Method Correcting for Quantitative PCR Efficiency Variations for Microbial Community Samples. *Appl Environ Microbiol* **2012**, *78* (12), 4481–4489. https://doi.org/10.1128/AEM.07878-11.

(22) Ruijter, J. M.; Ramakers, C.; Hoogaars, W. M. H.; Karlen, Y.; Bakker, O.; van den Hoff, M. J. B.; Moorman, A. F. M. Amplification Efficiency: Linking Baseline and Bias in the Analysis of Quantitative PCR Data. *Nucleic Acids Res* **2009**, *37* (6), e45. https://doi.org/10.1093/nar/gkp045.

(23) Edgar, R. C. SINTAX: A Simple Non-Bayesian Taxonomy Classifier for 16S and ITS Sequences. *bioRxiv* **2016**, 074161. https://doi.org/10.1101/074161.

(24) Edgar, R. C. SINTAX: a simple non-Bayesian taxonomy classifier for 16S and ITS sequences https://www.drive5.com/usearch/manual/sintax_downloads.html (accessed Dec 9, 2019). https://doi.org/10.1101/074161.

(25) Andersen, K. S.; Kirkegaard, R. H.; Karst, S. M.; Albertsen, M. Ampvis2: An R Package to Analyse and Visualise 16S RRNA Amplicon Data. *bioRxiv* **2018**, 299537. https://doi.org/10.1101/299537.

(26) R core team. *R: A Language and Environment for Statistical Computing.*; R Foundation for Statistical Computing: Vienna, Austria, 2014.

(27) Wickham, H. *Ggplot2: Elegant Graphics for Data Analysis*; Use R!; Springer-Verlag: New York, 2009. https://doi.org/10.1007/978-0-387-98141-3.

(28) Reddy, B.; Padovani, C.; Smart, N. R.; Rance, A. P.; Cook, A.; Milodowski, A.; Field, L.; Kemp, S.; Diomidis, N. Further Results on the in Situ Anaerobic Corrosion of Carbon Steel and Copper in Compacted Bentonite Exposed to Natural Opalinus Clay Porewater Containing Native Microbial Populations. *Materials and Corrosion* **2021**, *72* (1–2), 268–281. https://doi.org/10.1002/maco.202011785.

(29) Greaves, M. P.; Wilson, M. J. The Adsorption of Nucleic Acids by Montmorillonite. *Soil Biology and Biochemistry* **1969**, *1* (4), 317–323. https://doi.org/10.1016/0038-0717(69)90014-5.

# APPENDICES

## Alpha diversity data, Shannon and Simpson

| SampleID | Type | Location | Formulation | Density | Years | Module | Reads | Shannon | Simpson |
| --- | --- | --- | --- | --- | --- | --- | --- | --- | --- |
| 125Rep | Bentonite | Bulk interior | Block | 1.25 g cm^-3 | 0y | 0 | 2500 | 1.24323528 | 0.58871648 |
| 125ext | Bentonite | Bulk exterior | Block | 1.25 g cm^-3 | 0y | 0 | 2500 | 1.74956601 | 0.72174016 |
| 125int | Bentonite | Bulk interior | Block | 1.25 g cm^-3 | 0y | 0 | 2500 | 1.59328418 | 0.68546944 |
| 145Rep | Bentonite | Bulk interior | Block | 1.45 g cm^-3 | 0y | 0 | 2500 | 1.2720486 | 0.65405152 |
| 145ext | Bentonite | Bulk exterior | Block | 1.45 g cm^-3 | 0y | 0 | 2500 | 0.96335232 | 0.49898848 |
| 145int | Bentonite | Bulk interior | Block | 1.45 g cm^-3 | 0y | 0 | 2500 | 1.7274129 | 0.65131104 |
| BICA14 | Borehole | Borehole water | Borehole water | Borehole water | Ph1 2014 | 0 | 2500 | 1.82529236 | 0.7167536 |
| BICA17 | Borehole | Borehole water | Borehole water | Borehole water | Ph2 2017 | 0 | 2500 | 3.22326579 | 0.93008448 |
| BICA18 | Borehole | Borehole water | Borehole water | Borehole water | Ph3 2018 | 0 | 2500 | 3.51338793 | 0.9500288 |
| M13B1ext | Bentonite | Bulk exterior | Pellets | 1.45 g cm^-3 | 2.5y | 13 | 2500 | 3.69967629 | 0.95363136 |
| M13B1int | Bentonite | Bulk interior | Pellets | 1.45 g cm^-3 | 2.5y | 13 | 2500 | 3.22326976 | 0.91802144 |
| M13B2ext | Bentonite | Bulk exterior | Pellets | 1.45 g cm^-3 | 2.5y | 13 | 2500 | 3.43976332 | 0.94747744 |
| M13B2int | Bentonite | Bulk interior | Pellets | 1.45 g cm^-3 | 2.5y | 13 | 2500 | 3.51549978 | 0.94188384 |
| M13B3ext | Bentonite | Bulk exterior | Pellets | 1.45 g cm^-3 | 2.5y | 13 | 2500 | 2.55247115 | 0.86294752 |
| M13B3int | Bentonite | Bulk interior | Pellets | 1.45 g cm^-3 | 2.5y | 13 | 2500 | 2.13605746 | 0.80359104 |
| M13B4ext | Bentonite | Bulk exterior | Pellets | 1.45 g cm^-3 | 2.5y | 13 | 2500 | 2.5377897 | 0.88004512 |
| M13B4int | Bentonite | Bulk interior | Pellets | 1.45 g cm^-3 | 2.5y | 13 | 2500 | 2.33473781 | 0.83639712 |
| M13B5ext | Bentonite | Bulk exterior | Pellets | 1.45 g cm^-3 | 2.5y | 13 | 2500 | 2.12453255 | 0.82060352 |
| M13B5int | Bentonite | Bulk interior | Pellets | 1.45 g cm^-3 | 2.5y | 13 | 2500 | 2.89133031 | 0.89471296 |
| M13C10 | Bentonite | Coupon | Pellets | 1.45 g cm^-3 | 2.5y | 13 | 2500 | 2.97556113 | 0.88997504 |
| M13C10swab | Bentonite | Coupon | Pellets | 1.45 g cm^-3 | 2.5y | 13 | 2500 | 2.58053074 | 0.87794496 |
| M13C1 | Bentonite | Coupon | Pellets | 1.45 g cm^-3 | 2.5y | 13 | 2500 | 3.34079827 | 0.93308704 |
| M13C1swab | Bentonite | Coupon | Pellets | 1.45 g cm^-3 | 2.5y | 13 | 2500 | 2.20426363 | 0.8192432 |
| M13C4 | Bentonite | Coupon | Pellets | 1.45 g cm^-3 | 2.5y | 13 | 2500 | 2.29377689 | 0.64681024 |
| M13C4swab | Bentonite | Coupon | Pellets | 1.45 g cm^-3 | 2.5y | 13 | 2500 | 2.61033203 | 0.86089536 |
| M13C7 | Bentonite | Coupon | Pellets | 1.45 g cm^-3 | 2.5y | 13 | 2500 | 2.602865 | 0.87167296 |
| M13C7swab | Bentonite | Coupon | Pellets | 1.45 g cm^-3 | 2.5y | 13 | 2500 | 2.05361088 | 0.8163056 |
| M13F1ex | Filter | Filter outside | Pellets | 1.45 g cm^-3 | 2.5y | 13 | 2500 | 2.48099258 | 0.78272864 |
| M13F2ex | Filter | Filter outside | Pellets | 1.45 g cm^-3 | 2.5y | 13 | 2500 | 3.42852747 | 0.91865344 |
| M13F3in | Filter | Filter surface | Pellets | 1.45 g cm^-3 | 2.5y | 13 | 2500 | 2.13807361 | 0.75319712 |
| M13F4in | Filter | Filter BS | Pellets | 1.45 g cm^-3 | 2.5y | 13 | 2500 | 2.43439138 | 0.78021408 |
| M13F5in | Filter | Filter BS | Pellets | 1.45 g cm^-3 | 2.5y | 13 | 2500 | 2.13481554 | 0.71047968 |
| M13F6in | Filter | Filter BS | Pellets | 1.45 g cm^-3 | 2.5y | 13 | 2500 | 3.12590542 | 0.92673824 |
| M13H1 | Module | Module holes | Pellets | 1.45 g cm^-3 | 2.5y | 13 | 2500 | 3.49593251 | 0.9346992 |
| M13H2 | Module | Module holes | Pellets | 1.45 g cm^-3 | 2.5y | 13 | 2500 | 2.33056315 | 0.76898368 |
| M13H3 | Module | Module holes | Pellets | 1.45 g cm^-3 | 2.5y | 13 | 2500 | 2.34620658 | 0.7463104 |
| M13H4 | Module | Module holes | Pellets | 1.45 g cm^-3 | 2.5y | 13 | 2500 | 2.54999908 | 0.8185984 |
| M13H5 | Module | Module holes | Pellets | 1.45 g cm^-3 | 2.5y | 13 | 2500 | 3.35859084 | 0.90340032 |
| M13POI1deep | Core | BS cut | Pellets | 1.45 g cm^-3 | 2.5y | 13 | 2500 | 3.83614212 | 0.96409792 |
| M13POI1swab | Core | Core surface | Pellets | 1.45 g cm^-3 | 2.5y | 13 | 2500 | 2.18532874 | 0.75348736 |
| M13POI2deeper | Core | BS cut | Pellets | 1.45 g cm^-3 | 2.5y | 13 | 2500 | 3.21980629 | 0.92091264 |
| M13POI2swab | Core | BS swab | Pellets | 1.45 g cm^-3 | 2.5y | 13 | 2500 | 2.56878815 | 0.85393632 |
| M13POI3 | Core | BS swab | Pellets | 1.45 g cm^-3 | 2.5y | 13 | 2500 | 2.8580499 | 0.89527872 |
| M13POI3swab | Core | BS swab | Pellets | 1.45 g cm^-3 | 2.5y | 13 | 2500 | 2.72484152 | 0.84194432 |
| M13POI4swab | Core | BS swab | Pellets | 1.45 g cm^-3 | 2.5y | 13 | 2500 | 2.78203343 | 0.87652768 |
| M13POI5swab | Core | BS swab | Pellets | 1.45 g cm^-3 | 2.5y | 13 | 2500 | 2.56856168 | 0.87380352 |
| M13S1 | Module | Module surface | Pellets | 1.45 g cm^-3 | 2.5y | 13 | 2500 | 3.7243866 | 0.95497728 |
| M13S2 | Module | Module surface | Pellets | 1.45 g cm^-3 | 2.5y | 13 | 2500 | 3.94815252 | 0.96812032 |
| M13S3 | Module | Module surface | Pellets | 1.45 g cm^-3 | 2.5y | 13 | 2500 | 3.98149728 | 0.96835936 |
| M13S4 | Module | Module surface | Pellets | 1.45 g cm^-3 | 2.5y | 13 | 2500 | 3.95068748 | 0.96592 |
| M14B1ext | Bentonite | Bulk exterior | Block | 1.55 g cm^-3 | 2.5y | 14 | 2500 | 2.24409606 | 0.83913184 |
| M14B1int | Bentonite | Bulk interior | Block | 1.55 g cm^-3 | 2.5y | 14 | 2500 | 2.78201103 | 0.873664 |
| M14B2ext | Bentonite | Bulk exterior | Block | 1.55 g cm^-3 | 2.5y | 14 | 2500 | 3.12571577 | 0.90760288 |
| M14B2int | Bentonite | Bulk interior | Block | 1.55 g cm^-3 | 2.5y | 14 | 2500 | 2.35403998 | 0.8424496 |
| M14B3ext | Bentonite | Bulk exterior | Block | 1.55 g cm^-3 | 2.5y | 14 | 2500 | 2.37857611 | 0.85220896 |
| M14B3int | Bentonite | Bulk interior | Block | 1.55 g cm^-3 | 2.5y | 14 | 2500 | 2.23849162 | 0.82307808 |
| M14B4ext | Bentonite | Bulk exterior | Block | 1.55 g cm^-3 | 2.5y | 14 | 2500 | 4.10420234 | 0.96907488 |
| M14B4int | Bentonite | Bulk interior | Block | 1.55 g cm^-3 | 2.5y | 14 | 2500 | 3.53627755 | 0.92885856 |
| M14B5ext | Bentonite | Bulk exterior | Block | 1.55 g cm^-3 | 2.5y | 14 | 2500 | 4.11645386 | 0.9681696 |
| M14B5int | Bentonite | Bulk interior | Block | 1.55 g cm^-3 | 2.5y | 14 | 2500 | 4.03927367 | 0.96753728 |
| M14C11 | Bentonite | Coupon | Block | 1.55 g cm^-3 | 2.5y | 14 | 2500 | 2.85721152 | 0.90156064 |
| M14C11swab | Bentonite | Coupon | Block | 1.55 g cm^-3 | 2.5y | 14 | 2500 | 2.21888831 | 0.8479152 |
| M14C1 | Bentonite | Coupon | Block | 1.55 g cm^-3 | 2.5y | 14 | 2500 | 3.6491247 | 0.94811008 |
| M14C1swab | Bentonite | Coupon | Block | 1.55 g cm^-3 | 2.5y | 14 | 2500 | 1.85896976 | 0.8019856 |
| M14C4 | Bentonite | Coupon | Block | 1.55 g cm^-3 | 2.5y | 14 | 2500 | 2.7664323 | 0.89935296 |
| M14C4swab | Bentonite | Coupon | Block | 1.55 g cm^-3 | 2.5y | 14 | 2500 | 2.67561795 | 0.8847136 |
| M14C7 | Bentonite | Coupon | Block | 1.55 g cm^-3 | 2.5y | 14 | 2500 | 2.54792156 | 0.86102656 |
| M14C7swab | Bentonite | Coupon | Block | 1.55 g cm^-3 | 2.5y | 14 | 2500 | 2.2732736 | 0.83403136 |
| M14F1in | Filter | Filter surface | Block | 1.55 g cm^-3 | 2.5y | 14 | 2500 | 1.77610614 | 0.68581824 |
| M14F2in | Filter | Filter BS | Block | 1.55 g cm^-3 | 2.5y | 14 | 2500 | 2.39230712 | 0.81174944 |
| M14F3in | Filter | Filter BS | Block | 1.55 g cm^-3 | 2.5y | 14 | 2500 | 2.1106549 | 0.72657184 |
| M14F4in | Filter | Filter BS | Block | 1.55 g cm^-3 | 2.5y | 14 | 2500 | 2.0126917 | 0.71418624 |
| M14H1 | Module | Module holes | Block | 1.55 g cm^-3 | 2.5y | 14 | 2500 | 1.92955759 | 0.69873216 |
| M14H2 | Module | Module holes | Block | 1.55 g cm^-3 | 2.5y | 14 | 2500 | 2.87820288 | 0.85982464 |
| M14H3 | Module | Module holes | Block | 1.55 g cm^-3 | 2.5y | 14 | 2500 | 2.61545752 | 0.7927904 |
| M14H4 | Module | Module holes | Block | 1.55 g cm^-3 | 2.5y | 14 | 2500 | 3.79191104 | 0.95439616 |
| M14H5 | Module | Module holes | Block | 1.55 g cm^-3 | 2.5y | 14 | 2500 | 2.88552666 | 0.87216928 |
| M14POI1swab | Core | Core surface | Block | 1.55 g cm^-3 | 2.5y | 14 | 2500 | 1.56065679 | 0.63014816 |
| M14POI2swab | Core | Core shade | Block | 1.55 g cm^-3 | 2.5y | 14 | 2500 | 1.87477914 | 0.70301344 |
| M14POI3swab | Core | Core shade | Block | 1.55 g cm^-3 | 2.5y | 14 | 2500 | 2.24058387 | 0.75330496 |
| M14POI4swab | Core | Core shade | Block | 1.55 g cm^-3 | 2.5y | 14 | 2500 | 1.76856623 | 0.6586704 |
| M14POI5swab | Core | Core shade | Block | 1.55 g cm^-3 | 2.5y | 14 | 2500 | 1.98779449 | 0.72067648 |
| M14S1 | Module | Module surface | Block | 1.55 g cm^-3 | 2.5y | 14 | 2500 | 3.78831692 | 0.953104 |
| M14S2 | Module | Module surface | Block | 1.55 g cm^-3 | 2.5y | 14 | 2500 | 4.00135942 | 0.96715712 |
| M14S3 | Module | Module surface | Block | 1.55 g cm^-3 | 2.5y | 14 | 2500 | 4.04095958 | 0.97129952 |
| M14S4 | Module | Module surface | Block | 1.55 g cm^-3 | 2.5y | 14 | 2500 | 4.04386234 | 0.96929632 |
| M15B1ext | Bentonite | Bulk exterior | Block | 1.45 g cm^-3 | 2.5y | 15 | 2500 | 3.93616684 | 0.95428096 |
| M15B1int | Bentonite | Bulk interior | Block | 1.45 g cm^-3 | 2.5y | 15 | 2500 | 3.49912968 | 0.9478992 |
| M15B2ext | Bentonite | Bulk exterior | Block | 1.45 g cm^-3 | 2.5y | 15 | 2500 | 3.91159315 | 0.94401216 |
| M15B2int | Bentonite | Bulk interior | Block | 1.45 g cm^-3 | 2.5y | 15 | 2500 | 4.0611118 | 0.9635056 |
| M15B3ext | Bentonite | Bulk exterior | Block | 1.45 g cm^-3 | 2.5y | 15 | 2500 | 2.33660432 | 0.84474752 |
| M15B3int | Bentonite | Bulk interior | Block | 1.45 g cm^-3 | 2.5y | 15 | 2500 | 2.69982137 | 0.87663488 |
| M15B4ext | Bentonite | Bulk exterior | Block | 1.45 g cm^-3 | 2.5y | 15 | 2500 | 3.74367827 | 0.95874048 |
| M15B4int | Bentonite | Bulk interior | Block | 1.45 g cm^-3 | 2.5y | 15 | 2500 | 3.42388165 | 0.93186528 |
| M15B5ext | Bentonite | Bulk exterior | Block | 1.45 g cm^-3 | 2.5y | 15 | 2500 | 3.82150386 | 0.95731584 |
| M15B5int | Bentonite | Bulk interior | Block | 1.45 g cm^-3 | 2.5y | 15 | 2500 | 3.76805937 | 0.9603136 |
| M15C10 | Bentonite | Coupon | Block | 1.45 g cm^-3 | 2.5y | 15 | 2500 | 3.40011836 | 0.92434592 |
| M15C10swab | Bentonite | Coupon | Block | 1.45 g cm^-3 | 2.5y | 15 | 2500 | 2.22699615 | 0.82759808 |
| M15C1 | Bentonite | Coupon | Block | 1.45 g cm^-3 | 2.5y | 15 | 2500 | 2.73165944 | 0.874296 |
| M15C1swab | Bentonite | Coupon | Block | 1.45 g cm^-3 | 2.5y | 15 | 2500 | 2.49265768 | 0.85410048 |
| M15C4 | Bentonite | Coupon | Block | 1.45 g cm^-3 | 2.5y | 15 | 2500 | 2.47784073 | 0.87553536 |
| M15C4swab | Bentonite | Coupon | Block | 1.45 g cm^-3 | 2.5y | 15 | 2500 | 2.61008557 | 0.85543808 |
| M15C7 | Bentonite | Coupon | Block | 1.45 g cm^-3 | 2.5y | 15 | 2500 | 3.42351878 | 0.93005664 |
| M15C7swab | Bentonite | Coupon | Block | 1.45 g cm^-3 | 2.5y | 15 | 2500 | 2.26555824 | 0.80716352 |
| M15F1in | Filter | Filter BS | Block | 1.45 g cm^-3 | 2.5y | 15 | 2500 | 1.48818387 | 0.59771808 |
| M15F3in | Filter | Filter BS | Block | 1.45 g cm^-3 | 2.5y | 15 | 2500 | 2.68652297 | 0.89433056 |
| M15F4in | Filter | Filter BS | Block | 1.45 g cm^-3 | 2.5y | 15 | 2500 | 1.81712618 | 0.68857408 |
| M15F6in | Filter | Filter BS | Block | 1.45 g cm^-3 | 2.5y | 15 | 2500 | 2.11697364 | 0.72738048 |
| M15FnH1 | Filter | Filter outside | Block | 1.45 g cm^-3 | 2.5y | 15 | 2500 | 3.13414157 | 0.91361824 |
| M15FnH2 | Filter | Filter outside | Block | 1.45 g cm^-3 | 2.5y | 15 | 2500 | 3.61882111 | 0.94897792 |
| M15H1 | Module | Module holes | Block | 1.45 g cm^-3 | 2.5y | 15 | 2500 | 3.76827376 | 0.958504 |
| M15H2 | Module | Module holes | Block | 1.45 g cm^-3 | 2.5y | 15 | 2500 | 3.63485732 | 0.95206528 |
| M15H3 | Module | Module holes | Block | 1.45 g cm^-3 | 2.5y | 15 | 2500 | 3.33144938 | 0.915024 |
| M15H4 | Module | Module holes | Block | 1.45 g cm^-3 | 2.5y | 15 | 2500 | 3.24600302 | 0.89007136 |
| M15H5 | Module | Module holes | Block | 1.45 g cm^-3 | 2.5y | 15 | 2500 | 3.90022074 | 0.96286624 |
| M15POI1 | Filter | Filter outside | Block | 1.45 g cm^-3 | 2.5y | 15 | 2500 | 3.06638846 | 0.90573152 |
| M15POI1swab | Core | Core surface | Block | 1.45 g cm^-3 | 2.5y | 15 | 2500 | 3.13060149 | 0.9086336 |
| M15POI2bentonite | Core | Core shade | Block | 1.45 g cm^-3 | 2.5y | 15 | 2500 | 2.73728622 | 0.88126208 |
| M15POI3bentonite | Core | Core shade | Block | 1.45 g cm^-3 | 2.5y | 15 | 2500 | 1.23014464 | 0.53784544 |
| M15POI4bentonite | Core | BS swab | Block | 1.45 g cm^-3 | 2.5y | 15 | 2500 | 1.36561036 | 0.5797952 |
| M15POI5bentonite | Core | BS swab | Block | 1.45 g cm^-3 | 2.5y | 15 | 2500 | 1.57205485 | 0.61046464 |
| M15POI6bentonite | Core | BS swab | Block | 1.45 g cm^-3 | 2.5y | 15 | 2500 | 1.69101032 | 0.61956928 |
| M15S1 | Module | Module surface | Block | 1.45 g cm^-3 | 2.5y | 15 | 2500 | 3.9715747 | 0.96808672 |
| M15S3 | Module | Module surface | Block | 1.45 g cm^-3 | 2.5y | 15 | 2500 | 3.97125244 | 0.96371936 |
| M15S4 | Module | Module surface | Block | 1.45 g cm^-3 | 2.5y | 15 | 2500 | 3.87040612 | 0.96263296 |
| M16B1Ext | Bentonite | Bulk exterior | Pellets | 1.45 g cm^-3 | 1y | 16 | 2500 | 2.22924096 | 0.8254416 |
| M16B1Int | Bentonite | Bulk interior | Pellets | 1.45 g cm^-3 | 1y | 16 | 2500 | 3.50188992 | 0.93341664 |
| M16B2Ext | Bentonite | Bulk exterior | Pellets | 1.45 g cm^-3 | 1y | 16 | 2500 | 3.37874096 | 0.92831616 |
| M16B2Int | Bentonite | Bulk interior | Pellets | 1.45 g cm^-3 | 1y | 16 | 2500 | 2.49873063 | 0.84723872 |
| M16B3Ext | Bentonite | Bulk exterior | Pellets | 1.45 g cm^-3 | 1y | 16 | 2500 | 2.88976265 | 0.89424864 |
| M16B3Int | Bentonite | Bulk interior | Pellets | 1.45 g cm^-3 | 1y | 16 | 2500 | 2.98559345 | 0.89048256 |
| M16B4Ext | Bentonite | Bulk exterior | Pellets | 1.45 g cm^-3 | 1y | 16 | 2500 | 3.59144984 | 0.9449712 |
| M16B4Int | Bentonite | Bulk interior | Pellets | 1.45 g cm^-3 | 1y | 16 | 2500 | 3.54156731 | 0.94472064 |
| M16B5Ext | Bentonite | Bulk exterior | Pellets | 1.45 g cm^-3 | 1y | 16 | 2500 | 3.66444963 | 0.94323872 |
| M16B5Int | Bentonite | Bulk interior | Pellets | 1.45 g cm^-3 | 1y | 16 | 2500 | 2.40712343 | 0.82918656 |
| M16C10BM73 | Bentonite | Coupon | Pellets | 1.45 g cm^-3 | 1y | 16 | 2500 | 2.98567785 | 0.83094272 |
| M16C10swab | Bentonite | Coupon | Pellets | 1.45 g cm^-3 | 1y | 16 | 2500 | 2.64454988 | 0.89125152 |
| M16C1EDC73 | Bentonite | Coupon | Pellets | 1.45 g cm^-3 | 1y | 16 | 2500 | 2.45667645 | 0.86933632 |
| M16C1swab | Bentonite | Coupon | Pellets | 1.45 g cm^-3 | 1y | 16 | 2500 | 1.56268255 | 0.52327904 |
| M16C4CSC73 | Bentonite | Coupon | Pellets | 1.45 g cm^-3 | 1y | 16 | 2500 | 3.56660664 | 0.93657952 |
| M16C4swab | Bentonite | Coupon | Pellets | 1.45 g cm^-3 | 1y | 16 | 2500 | 2.39266827 | 0.84004256 |
| M16C7WM133 | Bentonite | Coupon | Pellets | 1.45 g cm^-3 | 1y | 16 | 2500 | 2.92355273 | 0.90628128 |
| M16C7swab | Bentonite | Coupon | Pellets | 1.45 g cm^-3 | 1y | 16 | 2500 | 2.35017963 | 0.8320656 |
| M16FBS1 | Filter | Filter BS | Pellets | 1.45 g cm^-3 | 1y | 16 | 2500 | 3.04617428 | 0.88847008 |
| M16FBS2 | Filter | Filter BS | Pellets | 1.45 g cm^-3 | 1y | 16 | 2500 | 2.867192 | 0.87835648 |
| M16FBS3 | Filter | Filter BS | Pellets | 1.45 g cm^-3 | 1y | 16 | 2500 | 3.38784086 | 0.93145344 |
| M16FBS4 | Filter | Filter BS | Pellets | 1.45 g cm^-3 | 1y | 16 | 2500 | 3.01611582 | 0.89385568 |
| M16FBS5 | Filter | Filter BS | Pellets | 1.45 g cm^-3 | 1y | 16 | 2500 | 2.79768096 | 0.89909184 |
| M16FInside1 | Filter | Filter surface | Pellets | 1.45 g cm^-3 | 1y | 16 | 2500 | 2.55521855 | 0.79833728 |
| M16H1 | Module | Module holes | Pellets | 1.45 g cm^-3 | 1y | 16 | 2500 | 3.99265346 | 0.9644272 |
| M16H2 | Module | Module holes | Pellets | 1.45 g cm^-3 | 1y | 16 | 2500 | 4.01403511 | 0.9651408 |
| M16H3 | Module | Module holes | Pellets | 1.45 g cm^-3 | 1y | 16 | 2500 | 4.01413445 | 0.96652032 |
| M16POI2 | Core | Core surface | Pellets | 1.45 g cm^-3 | 1y | 16 | 2500 | 2.12617494 | 0.73001408 |
| M16POI3 | Core | BS swab | Pellets | 1.45 g cm^-3 | 1y | 16 | 2500 | 3.08426049 | 0.9249344 |
| M16POI4 | Core | BS swab | Pellets | 1.45 g cm^-3 | 1y | 16 | 2500 | 2.93581467 | 0.89940448 |
| M16POI5 | Core | BS swab | Pellets | 1.45 g cm^-3 | 1y | 16 | 2500 | 2.68430145 | 0.84084288 |
| M16POI6 | Core | BS swab | Pellets | 1.45 g cm^-3 | 1y | 16 | 2500 | 2.65147407 | 0.88087104 |
| M16POI7 | Core | BS swab | Pellets | 1.45 g cm^-3 | 1y | 16 | 2500 | 2.73667953 | 0.889152 |
| M16S1 | Module | Module surface | Pellets | 1.45 g cm^-3 | 1y | 16 | 2500 | 3.48582194 | 0.90746784 |
| M16S2 | Module | Module surface | Pellets | 1.45 g cm^-3 | 1y | 16 | 2500 | 4.14743913 | 0.96857376 |
| M16S3 | Module | Module surface | Pellets | 1.45 g cm^-3 | 1y | 16 | 2500 | 4.07518333 | 0.96881472 |
| M17B1Ext | Bentonite | Bulk exterior | Block | 1.25 g cm^-3 | 1y | 17 | 2500 | 2.70738013 | 0.83689792 |
| M17B1Int | Bentonite | Bulk interior | Block | 1.25 g cm^-3 | 1y | 17 | 2500 | 2.66708876 | 0.82206464 |
| M17B2Ext | Bentonite | Bulk exterior | Block | 1.25 g cm^-3 | 1y | 17 | 2500 | 2.78942241 | 0.85228576 |
| M17B2Int | Bentonite | Bulk interior | Block | 1.25 g cm^-3 | 1y | 17 | 2500 | 2.88285916 | 0.90304768 |
| M17B3Ext | Bentonite | Bulk exterior | Block | 1.25 g cm^-3 | 1y | 17 | 2500 | 2.83652796 | 0.90331648 |
| M17B3Int | Bentonite | Bulk interior | Block | 1.25 g cm^-3 | 1y | 17 | 2500 | 2.87523885 | 0.83711264 |
| M17B4Ext | Bentonite | Bulk exterior | Block | 1.25 g cm^-3 | 1y | 17 | 2500 | 2.93953838 | 0.86588928 |
| M17B4Int | Bentonite | Bulk interior | Block | 1.25 g cm^-3 | 1y | 17 | 2500 | 3.02091198 | 0.90264832 |
| M17B5Ext | Bentonite | Bulk exterior | Block | 1.25 g cm^-3 | 1y | 17 | 2500 | 2.93360942 | 0.89821408 |
| M17B5Int | Bentonite | Bulk interior | Block | 1.25 g cm^-3 | 1y | 17 | 2500 | 2.53712324 | 0.80579424 |
| M17C10BM76 | Bentonite | Coupon | Block | 1.25 g cm^-3 | 1y | 17 | 2500 | 2.25475333 | 0.79321184 |
| M17C10swab | Bentonite | Coupon | Block | 1.25 g cm^-3 | 1y | 17 | 2500 | 1.61938568 | 0.63565312 |
| M17C1EDC76 | Bentonite | Coupon | Block | 1.25 g cm^-3 | 1y | 17 | 2500 | 2.68374527 | 0.88368192 |
| M17C1swab | Bentonite | Coupon | Block | 1.25 g cm^-3 | 1y | 17 | 2500 | 2.99470786 | 0.91127904 |
| M17C4CSC76 | Bentonite | Coupon | Block | 1.25 g cm^-3 | 1y | 17 | 2500 | 2.55478941 | 0.88010208 |
| M17C4swab | Bentonite | Coupon | Block | 1.25 g cm^-3 | 1y | 17 | 2500 | 1.81452409 | 0.6687008 |
| M17C7WM141 | Bentonite | Coupon | Block | 1.25 g cm^-3 | 1y | 17 | 2500 | 2.38590409 | 0.83107008 |
| M17C7swab | Bentonite | Coupon | Block | 1.25 g cm^-3 | 1y | 17 | 2500 | 3.07167935 | 0.92394784 |
| M17F1 | Filter | Filter surface | Block | 1.25 g cm^-3 | 1y | 17 | 2500 | 3.01585038 | 0.90981952 |
| M17FBS1 | Filter | Filter BS | Block | 1.25 g cm^-3 | 1y | 17 | 2500 | 2.98387285 | 0.91470464 |
| M17FBS2 | Filter | Filter BS | Block | 1.25 g cm^-3 | 1y | 17 | 2500 | 3.15361523 | 0.92690208 |
| M17FBS3 | Filter | Filter BS | Block | 1.25 g cm^-3 | 1y | 17 | 2500 | 3.12874165 | 0.93578304 |
| M17FBS4 | Filter | Filter BS | Block | 1.25 g cm^-3 | 1y | 17 | 2500 | 2.47318644 | 0.84214784 |
| M17H1 | Module | Module holes | Block | 1.25 g cm^-3 | 1y | 17 | 2500 | 3.47068534 | 0.90878336 |
| M17H2 | Module | Module holes | Block | 1.25 g cm^-3 | 1y | 17 | 2500 | 4.04252251 | 0.96415008 |
| M17H3 | Module | Module holes | Block | 1.25 g cm^-3 | 1y | 17 | 2500 | 3.42747583 | 0.90791744 |
| M17POI3 | Core | BS swab | Block | 1.25 g cm^-3 | 1y | 17 | 2500 | 2.5732504 | 0.84337728 |
| M17POI3cut11 | Core | BS cut | Block | 1.25 g cm^-3 | 1y | 17 | 2500 | 2.56052343 | 0.85558336 |
| M17POI3cut12 | Core | BS cut | Block | 1.25 g cm^-3 | 1y | 17 | 2500 | 2.6787536 | 0.86706528 |
| M17POI3cut2 | Core | BS cut | Block | 1.25 g cm^-3 | 1y | 17 | 2500 | 2.16897056 | 0.76040832 |
| M17POI4 | Core | BS swab | Block | 1.25 g cm^-3 | 1y | 17 | 2500 | 3.32543853 | 0.94154912 |
| M17POI5 | Core | BS swab | Block | 1.25 g cm^-3 | 1y | 17 | 2500 | 2.27501712 | 0.76626016 |
| M17POI6 | Core | BS swab | Block | 1.25 g cm^-3 | 1y | 17 | 2500 | 2.6982171 | 0.87750816 |
| M17POI7 | Core | Core surface | Block | 1.25 g cm^-3 | 1y | 17 | 2500 | 3.06517025 | 0.91878592 |
| M17S1 | Module | Module surface | Block | 1.25 g cm^-3 | 1y | 17 | 2500 | 3.26665162 | 0.87793536 |
| M17S2 | Module | Module surface | Block | 1.25 g cm^-3 | 1y | 17 | 2500 | 3.65318841 | 0.917264 |
| M17S3 | Module | Module surface | Block | 1.25 g cm^-3 | 1y | 17 | 2500 | 3.50809587 | 0.90418656 |
| M18B1Ext | Bentonite | Bulk exterior | Block | 1.45 g cm^-3 | 1y | 18 | 2500 | 2.47865185 | 0.86795648 |
| M18B1Int | Bentonite | Bulk interior | Block | 1.45 g cm^-3 | 1y | 18 | 2500 | 1.88254288 | 0.64084512 |
| M18B2Ext | Bentonite | Bulk exterior | Block | 1.45 g cm^-3 | 1y | 18 | 2500 | 3.25991677 | 0.90832192 |
| M18B2Int | Bentonite | Bulk interior | Block | 1.45 g cm^-3 | 1y | 18 | 2500 | 2.93386487 | 0.8982288 |
| M18B3Ext | Bentonite | Bulk exterior | Block | 1.45 g cm^-3 | 1y | 18 | 2500 | 3.72061893 | 0.946504 |
| M18B3Int | Bentonite | Bulk interior | Block | 1.45 g cm^-3 | 1y | 18 | 2500 | 3.36104219 | 0.9181632 |
| M18B4Ext | Bentonite | Bulk exterior | Block | 1.45 g cm^-3 | 1y | 18 | 2500 | 3.06121079 | 0.8773984 |
| M18B4Int | Bentonite | Bulk interior | Block | 1.45 g cm^-3 | 1y | 18 | 2500 | 3.29617115 | 0.9391856 |
| M18B5Ext | Bentonite | Bulk exterior | Block | 1.45 g cm^-3 | 1y | 18 | 2500 | 2.44822797 | 0.85942752 |
| M18B5Int | Bentonite | Bulk interior | Block | 1.45 g cm^-3 | 1y | 18 | 2500 | 3.15812114 | 0.8751776 |
| M18C10swab | Bentonite | Coupon | Block | 1.45 g cm^-3 | 1y | 18 | 2500 | 1.93638827 | 0.77060608 |
| M18C1EDC83 | Bentonite | Coupon | Block | 1.45 g cm^-3 | 1y | 18 | 2500 | 3.2431078 | 0.89435168 |
| M18C1swab | Bentonite | Coupon | Block | 1.45 g cm^-3 | 1y | 18 | 2500 | 2.05980345 | 0.77700768 |
| M18C4CSC83 | Bentonite | Coupon | Block | 1.45 g cm^-3 | 1y | 18 | 2500 | 2.83639592 | 0.90705664 |
| M18C4swab | Bentonite | Coupon | Block | 1.45 g cm^-3 | 1y | 18 | 2500 | 3.05826721 | 0.88769728 |
| M18C7WM144 | Bentonite | Coupon | Block | 1.45 g cm^-3 | 1y | 18 | 2500 | 2.48203628 | 0.7982192 |
| M18C7swab | Bentonite | Coupon | Block | 1.45 g cm^-3 | 1y | 18 | 2500 | 2.54993459 | 0.838456 |
| M18F1 | Filter | Filter surface | Block | 1.45 g cm^-3 | 1y | 18 | 2500 | 2.93890201 | 0.90605984 |
| M18FBS1 | Filter | Filter BS | Block | 1.45 g cm^-3 | 1y | 18 | 2500 | 2.56335232 | 0.82180128 |
| M18FBS2 | Filter | Filter BS | Block | 1.45 g cm^-3 | 1y | 18 | 2500 | 2.88971202 | 0.90285376 |
| M18FBS3 | Filter | Filter BS | Block | 1.45 g cm^-3 | 1y | 18 | 2500 | 1.93384282 | 0.64086336 |
| M18FBS4 | Filter | Filter BS | Block | 1.45 g cm^-3 | 1y | 18 | 2500 | 2.87256207 | 0.88972256 |
| M18FExtra1 | Filter | Filter outside | Block | 1.45 g cm^-3 | 1y | 18 | 2500 | 2.31475395 | 0.84096 |
| M18H1 | Module | Module holes | Block | 1.45 g cm^-3 | 1y | 18 | 2500 | 3.21849208 | 0.90139552 |
| M18H2 | Module | Module holes | Block | 1.45 g cm^-3 | 1y | 18 | 2500 | 2.66249398 | 0.84085312 |
| M18POI1 | Core | Core surface | Block | 1.45 g cm^-3 | 1y | 18 | 2500 | 2.98745776 | 0.90880896 |
| M18POI2 | Core | BS swab | Block | 1.45 g cm^-3 | 1y | 18 | 2500 | 2.42017697 | 0.80484416 |
| M18POI2cut | Core | BS cut | Block | 1.45 g cm^-3 | 1y | 18 | 2500 | 2.08950111 | 0.77840384 |
| M18POI3 | Core | BS swab | Block | 1.45 g cm^-3 | 1y | 18 | 2500 | 3.02270481 | 0.92211136 |
| M18POI3cut | Core | BS cut | Block | 1.45 g cm^-3 | 1y | 18 | 2500 | 2.54324071 | 0.88320608 |
| M18POI4 | Core | BS swab | Block | 1.45 g cm^-3 | 1y | 18 | 2500 | 3.16204576 | 0.92468704 |
| M18POI4cut | Core | BS cut | Block | 1.45 g cm^-3 | 1y | 18 | 2500 | 2.17410472 | 0.79686112 |
| M18POI5 | Core | Core shade | Block | 1.45 g cm^-3 | 1y | 18 | 2500 | 2.93371729 | 0.90171136 |
| M18POI6 | Core | Core shade | Block | 1.45 g cm^-3 | 1y | 18 | 2500 | 2.65784588 | 0.88702624 |
| M18POI6cut | Core | Core shade | Block | 1.45 g cm^-3 | 1y | 18 | 2500 | 1.65352208 | 0.74223616 |
| M18S1 | Module | Module surface | Block | 1.45 g cm^-3 | 1y | 18 | 2500 | 2.20944765 | 0.80736032 |
| M18S2 | Module | Module surface | Block | 1.45 g cm^-3 | 1y | 18 | 2500 | 3.01865804 | 0.85041216 |
| M18S3 | Module | Module surface | Block | 1.45 g cm^-3 | 1y | 18 | 2500 | 3.36085374 | 0.8949936 |
| M4B1ExtRep | Bentonite | Bulk exterior | Block | 1.45 g cm^-3 | 5.5y | 4 | 2500 | 3.29582977 | 0.9288432 |
| M4B1Ext | Bentonite | Bulk exterior | Block | 1.45 g cm^-3 | 5.5y | 4 | 2500 | 3.8925648 | 0.95507072 |
| M4B1IntRep | Bentonite | Bulk interior | Block | 1.45 g cm^-3 | 5.5y | 4 | 2500 | 3.65750237 | 0.93605568 |
| M4B1Int | Bentonite | Bulk interior | Block | 1.45 g cm^-3 | 5.5y | 4 | 2500 | 3.51397008 | 0.92951872 |
| M4B2Ext | Bentonite | Bulk exterior | Block | 1.45 g cm^-3 | 5.5y | 4 | 2500 | 3.05094146 | 0.90783808 |
| M4B2Int | Bentonite | Bulk interior | Block | 1.45 g cm^-3 | 5.5y | 4 | 2500 | 3.59584883 | 0.9492864 |
| M4B3Ext | Bentonite | Bulk exterior | Block | 1.45 g cm^-3 | 5.5y | 4 | 2500 | 3.24564561 | 0.91944032 |
| M4B3Int | Bentonite | Bulk interior | Block | 1.45 g cm^-3 | 5.5y | 4 | 2500 | 2.00070973 | 0.77684544 |
| M4B4Ext | Bentonite | Bulk exterior | Block | 1.45 g cm^-3 | 5.5y | 4 | 2500 | 3.02870955 | 0.9208016 |
| M4B4Int | Bentonite | Bulk interior | Block | 1.45 g cm^-3 | 5.5y | 4 | 2500 | 3.48600475 | 0.94563808 |
| M4B5Ext | Bentonite | Bulk exterior | Block | 1.45 g cm^-3 | 5.5y | 4 | 2500 | 3.30312137 | 0.9272352 |
| M4B5Int | Bentonite | Bulk interior | Block | 1.45 g cm^-3 | 5.5y | 4 | 2500 | 3.21169243 | 0.92692512 |
| M4C10BM28 | Bentonite | Coupon | Block | 1.45 g cm^-3 | 5.5y | 4 | 2500 | 3.53336164 | 0.89528192 |
| M4C10swab | Bentonite | Coupon | Block | 1.45 g cm^-3 | 5.5y | 4 | 2500 | 1.62876763 | 0.56244352 |
| M4C1SW5A | Bentonite | Coupon | Block | 1.45 g cm^-3 | 5.5y | 4 | 2500 | 3.54442391 | 0.88255552 |
| M4C1swab | Bentonite | Coupon | Block | 1.45 g cm^-3 | 5.5y | 4 | 2500 | 1.50781664 | 0.51311008 |
| M4C4WM45 | Bentonite | Coupon | Block | 1.45 g cm^-3 | 5.5y | 4 | 2500 | 3.62934882 | 0.9001408 |
| M4C4swab | Bentonite | Coupon | Block | 1.45 g cm^-3 | 5.5y | 4 | 2500 | 1.72987771 | 0.57286656 |
| M4C7swab | Bentonite | Coupon | Block | 1.45 g cm^-3 | 5.5y | 4 | 2500 | 1.4986083 | 0.53417728 |
| M4CleanCheeseKnifeSpatula | Control | Contamination control | Contamination control | Contamination control | 5.5y | 4 | 2500 | 1.65151387 | 0.53265056 |
| M4F1 | Filter | Filter surface | Block | 1.45 g cm^-3 | 5.5y | 4 | 2500 | 2.29525679 | 0.77696512 |
| M4FBS1 | Filter | Filter BS | Block | 1.45 g cm^-3 | 5.5y | 4 | 2500 | 1.82442856 | 0.60712384 |
| M4FBS2 | Filter | Filter BS | Block | 1.45 g cm^-3 | 5.5y | 4 | 2500 | 2.46950617 | 0.85635168 |
| M4FBS3 | Filter | Filter BS | Block | 1.45 g cm^-3 | 5.5y | 4 | 2500 | 2.74110099 | 0.90359648 |
| M4FBS4 | Filter | Filter BS | Block | 1.45 g cm^-3 | 5.5y | 4 | 2500 | 2.89137871 | 0.91075776 |
| M4FPOI1 | Filter | Filter BS | Block | 1.45 g cm^-3 | 5.5y | 4 | 2500 | 1.73327504 | 0.604208 |
| M4FPOI2 | Filter | Filter BS | Block | 1.45 g cm^-3 | 5.5y | 4 | 2500 | 2.03946029 | 0.72928704 |
| M4H1 | Module | Module holes | Block | 1.45 g cm^-3 | 5.5y | 4 | 2500 | 3.71739434 | 0.940824 |
| M4H2 | Module | Module holes | Block | 1.45 g cm^-3 | 5.5y | 4 | 2500 | 3.4226412 | 0.89825216 |
| M4H3 | Module | Module holes | Block | 1.45 g cm^-3 | 5.5y | 4 | 2500 | 3.7464418 | 0.94074208 |
| M4POI10 | Core | BS swab | Block | 1.45 g cm^-3 | 5.5y | 4 | 2500 | 1.95088354 | 0.63795232 |
| M4POI11 | Core | BS swab | Block | 1.45 g cm^-3 | 5.5y | 4 | 2500 | 2.01074794 | 0.72023008 |
| M4POI12cut | Core | BS cut | Block | 1.45 g cm^-3 | 5.5y | 4 | 2500 | 1.84115077 | 0.65336064 |
| M4POI3 | Core | Core surface | Block | 1.45 g cm^-3 | 5.5y | 4 | 2500 | 1.93454951 | 0.69155328 |
| M4POI4 | Core | BS swab | Block | 1.45 g cm^-3 | 5.5y | 4 | 2500 | 3.33822518 | 0.94154528 |
| M4POI4cut | Core | BS cut | Block | 1.45 g cm^-3 | 5.5y | 4 | 2500 | 3.38617078 | 0.94022592 |
| M4POI5 | Core | Core shade | Block | 1.45 g cm^-3 | 5.5y | 4 | 2500 | 3.20533981 | 0.92240608 |
| M4POI6 | Core | Core shade | Block | 1.45 g cm^-3 | 5.5y | 4 | 2500 | 2.38815417 | 0.80805248 |
| M4POI6cut | Core | Core shade | Block | 1.45 g cm^-3 | 5.5y | 4 | 2500 | 3.71299896 | 0.93957856 |
| M4POI7 | Core | BS swab | Block | 1.45 g cm^-3 | 5.5y | 4 | 2500 | 2.04472974 | 0.75814432 |
| M4POI7cut | Core | BS cut | Block | 1.45 g cm^-3 | 5.5y | 4 | 2500 | 2.8846402 | 0.84260896 |
| M4POI8 | Core | Core shade | Block | 1.45 g cm^-3 | 5.5y | 4 | 2500 | 2.31097742 | 0.79635904 |
| M4POI8cut | Core | Core shade | Block | 1.45 g cm^-3 | 5.5y | 4 | 2500 | 2.16437692 | 0.7076592 |
| M4POI9 | Core | BS swab | Block | 1.45 g cm^-3 | 5.5y | 4 | 2500 | 2.33085713 | 0.75693632 |
| M4S1 | Module | Module surface | Block | 1.45 g cm^-3 | 5.5y | 4 | 2500 | 4.05847488 | 0.96761184 |
| M4S2 | Module | Module surface | Block | 1.45 g cm^-3 | 5.5y | 4 | 2500 | 4.17049317 | 0.97191808 |
| M4S3 | Module | Module surface | Block | 1.45 g cm^-3 | 5.5y | 4 | 2500 | 4.22163156 | 0.97240576 |
| M5B1Ext | Bentonite | Bulk exterior | Block | 1.25 g cm^-3 | 5.5y | 5 | 2500 | 3.1039385 | 0.8678112 |
| M5B1Int | Bentonite | Bulk interior | Block | 1.25 g cm^-3 | 5.5y | 5 | 2500 | 3.31711886 | 0.8847264 |
| M5B2Ext | Bentonite | Bulk exterior | Block | 1.25 g cm^-3 | 5.5y | 5 | 2500 | 2.63849996 | 0.88538048 |
| M5B2Int | Bentonite | Bulk interior | Block | 1.25 g cm^-3 | 5.5y | 5 | 2500 | 2.95075452 | 0.8712944 |
| M5B3Ext | Bentonite | Bulk exterior | Block | 1.25 g cm^-3 | 5.5y | 5 | 2500 | 2.69527349 | 0.8982592 |
| M5B3Int | Bentonite | Bulk interior | Block | 1.25 g cm^-3 | 5.5y | 5 | 2500 | 2.82786975 | 0.82662304 |
| M5B4Ext | Bentonite | Bulk exterior | Block | 1.25 g cm^-3 | 5.5y | 5 | 2500 | 2.9254255 | 0.84626752 |
| M5B4Int | Bentonite | Bulk interior | Block | 1.25 g cm^-3 | 5.5y | 5 | 2500 | 2.83893367 | 0.87047808 |
| M5B5Ext | Bentonite | Bulk exterior | Block | 1.25 g cm^-3 | 5.5y | 5 | 2500 | 2.33273114 | 0.78908352 |
| M5B5Int | Bentonite | Bulk interior | Block | 1.25 g cm^-3 | 5.5y | 5 | 2500 | 3.10679382 | 0.85332128 |
| M5C10BM17 | Bentonite | Coupon | Block | 1.25 g cm^-3 | 5.5y | 5 | 2500 | 3.70902138 | 0.94198816 |
| M5C10swab | Bentonite | Coupon | Block | 1.25 g cm^-3 | 5.5y | 5 | 2500 | 3.17296593 | 0.9305136 |
| M5C1DW2A | Bentonite | Coupon | Block | 1.25 g cm^-3 | 5.5y | 5 | 2500 | 3.18351156 | 0.89858112 |
| M5C1swab | Bentonite | Coupon | Block | 1.25 g cm^-3 | 5.5y | 5 | 2500 | 2.97492114 | 0.91476352 |
| M5C4F3 | Bentonite | Coupon | Block | 1.25 g cm^-3 | 5.5y | 5 | 2500 | 3.45393034 | 0.92182176 |
| M5C4swab | Bentonite | Coupon | Block | 1.25 g cm^-3 | 5.5y | 5 | 2500 | 3.00826165 | 0.90322048 |
| M5C7WM21 | Bentonite | Coupon | Block | 1.25 g cm^-3 | 5.5y | 5 | 2500 | 3.71123158 | 0.93888576 |
| M5C7swab | Bentonite | Coupon | Block | 1.25 g cm^-3 | 5.5y | 5 | 2500 | 3.04180933 | 0.91128704 |
| M5F1 | Filter | Filter surface | Block | 1.25 g cm^-3 | 5.5y | 5 | 2500 | 3.17989266 | 0.92309088 |
| M5FBS1 | Filter | Filter BS | Block | 1.25 g cm^-3 | 5.5y | 5 | 2500 | 2.72335917 | 0.86245056 |
| M5FBS2 | Filter | Filter BS | Block | 1.25 g cm^-3 | 5.5y | 5 | 2500 | 2.02700666 | 0.7422368 |
| M5FBS3 | Filter | Filter BS | Block | 1.25 g cm^-3 | 5.5y | 5 | 2500 | 2.15616438 | 0.79672288 |
| M5FPOI1 | Filter | Filter BS | Block | 1.25 g cm^-3 | 5.5y | 5 | 2500 | 1.54817318 | 0.66133056 |
| M5FPOI2 | Filter | Filter BS | Block | 1.25 g cm^-3 | 5.5y | 5 | 2500 | 3.21138167 | 0.92761408 |
| M5FPOI3 | Filter | Filter BS | Block | 1.25 g cm^-3 | 5.5y | 5 | 2500 | 2.01341707 | 0.74638272 |
| M5H1 | Module | Module holes | Block | 1.25 g cm^-3 | 5.5y | 5 | 2500 | 3.70006798 | 0.93176128 |
| M5H2 | Module | Module holes | Block | 1.25 g cm^-3 | 5.5y | 5 | 2500 | 3.77968111 | 0.94131776 |
| M5H3 | Module | Module holes | Block | 1.25 g cm^-3 | 5.5y | 5 | 2500 | 3.55422119 | 0.91053824 |
| M5POI1 | Core | BS swab | Block | 1.25 g cm^-3 | 5.5y | 5 | 2500 | 2.80770727 | 0.88711904 |
| M5POI1cut | Core | BS cut | Block | 1.25 g cm^-3 | 5.5y | 5 | 2500 | 2.99991887 | 0.92545216 |
| M5POI2 | Core | BS swab | Block | 1.25 g cm^-3 | 5.5y | 5 | 2500 | 1.69892468 | 0.72766656 |
| M5POI2cut | Core | BS cut | Block | 1.25 g cm^-3 | 5.5y | 5 | 2500 | 2.39916567 | 0.85950496 |
| M5POI3 | Core | BS swab | Block | 1.25 g cm^-3 | 5.5y | 5 | 2500 | 2.32434866 | 0.79613632 |
| M5POI4 | Core | BS swab | Block | 1.25 g cm^-3 | 5.5y | 5 | 2500 | 2.02537353 | 0.8067344 |
| M5POI4cut | Core | BS cut | Block | 1.25 g cm^-3 | 5.5y | 5 | 2500 | 2.58687264 | 0.85380928 |
| M5POI5 | Core | BS swab | Block | 1.25 g cm^-3 | 5.5y | 5 | 2500 | 1.55634752 | 0.67888384 |
| M5POI5cut | Core | BS cut | Block | 1.25 g cm^-3 | 5.5y | 5 | 2500 | 1.89570459 | 0.75027744 |
| M5POI6 | Core | BS swab | Block | 1.25 g cm^-3 | 5.5y | 5 | 2500 | 3.44991653 | 0.93587968 |
| M5POI7 | Core | BS swab | Block | 1.25 g cm^-3 | 5.5y | 5 | 2500 | 2.13949817 | 0.783616 |
| M5POI8 | Core | Core surface | Block | 1.25 g cm^-3 | 5.5y | 5 | 2500 | 2.31930606 | 0.83081664 |
| M5POI8b | Core | Core surface | Block | 1.25 g cm^-3 | 5.5y | 5 | 2500 | 3.31839077 | 0.94225568 |
| M5S1 | Module | Module surface | Block | 1.25 g cm^-3 | 5.5y | 5 | 2500 | 4.15002432 | 0.96360384 |
| M5S2 | Module | Module surface | Block | 1.25 g cm^-3 | 5.5y | 5 | 2500 | 3.95253276 | 0.9533728 |
| M5S3 | Module | Module surface | Block | 1.25 g cm^-3 | 5.5y | 5 | 2500 | 4.08795406 | 0.96468128 |
| M6B1Ext | Bentonite | Bulk exterior | Block | 1.55 g cm^-3 | 5.5y | 6 | 2500 | 2.80335037 | 0.88491968 |
| M6B1Int | Bentonite | Bulk interior | Block | 1.55 g cm^-3 | 5.5y | 6 | 2500 | 3.79892049 | 0.95175392 |
| M6B2Ext | Bentonite | Bulk exterior | Block | 1.55 g cm^-3 | 5.5y | 6 | 2500 | 3.06681706 | 0.91991552 |
| M6B2Int | Bentonite | Bulk interior | Block | 1.55 g cm^-3 | 5.5y | 6 | 2500 | 3.22475985 | 0.9060128 |
| M6B3Ext | Bentonite | Bulk exterior | Block | 1.55 g cm^-3 | 5.5y | 6 | 2500 | 1.62920837 | 0.45818976 |
| M6B3Int | Bentonite | Bulk interior | Block | 1.55 g cm^-3 | 5.5y | 6 | 2500 | 2.69198315 | 0.86440704 |
| M6B4Ext | Bentonite | Bulk exterior | Block | 1.55 g cm^-3 | 5.5y | 6 | 2500 | 3.70879948 | 0.95678496 |
| M6B4Int | Bentonite | Bulk interior | Block | 1.55 g cm^-3 | 5.5y | 6 | 2500 | 3.48660598 | 0.9183632 |
| M6B5Ext | Bentonite | Bulk exterior | Block | 1.55 g cm^-3 | 5.5y | 6 | 2500 | 3.39371144 | 0.89549344 |
| M6B5Int | Bentonite | Bulk interior | Block | 1.55 g cm^-3 | 5.5y | 6 | 2500 | 3.23300818 | 0.90335872 |
| M6C10BM36 | Bentonite | Coupon | Block | 1.55 g cm^-3 | 5.5y | 6 | 2500 | 4.38278705 | 0.94464896 |
| M6C10swab | Bentonite | Coupon | Block | 1.55 g cm^-3 | 5.5y | 6 | 2500 | 1.87851449 | 0.7661264 |
| M6C1SW7A | Bentonite | Coupon | Block | 1.55 g cm^-3 | 5.5y | 6 | 2500 | 3.46916559 | 0.90518144 |
| M6C1swab | Bentonite | Coupon | Block | 1.55 g cm^-3 | 5.5y | 6 | 2500 | 2.60935824 | 0.83841536 |
| M6C4WM63 | Bentonite | Coupon | Block | 1.55 g cm^-3 | 5.5y | 6 | 2500 | 2.65876207 | 0.82033664 |
| M6C4swab | Bentonite | Coupon | Block | 1.55 g cm^-3 | 5.5y | 6 | 2500 | 2.55355785 | 0.87770272 |
| M6C7WM55 | Bentonite | Coupon | Block | 1.55 g cm^-3 | 5.5y | 6 | 2500 | 1.94137147 | 0.7568944 |
| M6C7swab | Bentonite | Coupon | Block | 1.55 g cm^-3 | 5.5y | 6 | 2500 | 2.98956291 | 0.92027328 |
| M6F1 | Filter | Filter surface | Block | 1.55 g cm^-3 | 5.5y | 6 | 2500 | 2.6494828 | 0.82377152 |
| M6FBS1 | Filter | Filter BS | Block | 1.55 g cm^-3 | 5.5y | 6 | 2500 | 2.85247034 | 0.90332256 |
| M6FBS2 | Filter | Filter BS | Block | 1.55 g cm^-3 | 5.5y | 6 | 2500 | 3.17397793 | 0.9399488 |
| M6FBS3 | Filter | Filter BS | Block | 1.55 g cm^-3 | 5.5y | 6 | 2500 | 2.62757651 | 0.84730656 |
| M6FPOI1 | Filter | Filter BS | Block | 1.55 g cm^-3 | 5.5y | 6 | 2500 | 2.62049 | 0.82761696 |
| M6FPOI2 | Filter | Filter BS | Block | 1.55 g cm^-3 | 5.5y | 6 | 2500 | 2.72886773 | 0.86237216 |
| M6FPOI3 | Filter | Filter BS | Block | 1.55 g cm^-3 | 5.5y | 6 | 2500 | 2.5598722 | 0.84921888 |
| M6FPOI4 | Filter | Filter BS | Block | 1.55 g cm^-3 | 5.5y | 6 | 2500 | 2.40534159 | 0.81662464 |
| M6H1 | Module | Module holes | Block | 1.55 g cm^-3 | 5.5y | 6 | 2500 | 2.57583699 | 0.83887904 |
| M6H2 | Module | Module holes | Block | 1.55 g cm^-3 | 5.5y | 6 | 2500 | 2.88852968 | 0.87355456 |
| M6H3 | Module | Module holes | Block | 1.55 g cm^-3 | 5.5y | 6 | 2500 | 2.897771 | 0.84936288 |
| M6POI10 | Core | Core shade | Block | 1.55 g cm^-3 | 5.5y | 6 | 2500 | 3.14459918 | 0.92286048 |
| M6POI10cut | Core | Core shade | Block | 1.55 g cm^-3 | 5.5y | 6 | 2500 | 1.98259284 | 0.75603872 |
| M6POI11 | Core | Core shade | Block | 1.55 g cm^-3 | 5.5y | 6 | 2500 | 2.41796912 | 0.85132672 |
| M6POI12 | Core | Core shade | Block | 1.55 g cm^-3 | 5.5y | 6 | 2500 | 2.3617795 | 0.81102016 |
| M6POI12cut | Core | Core shade | Block | 1.55 g cm^-3 | 5.5y | 6 | 2500 | 3.08245728 | 0.8892992 |
| M6POI3 | Core | Core surface | Block | 1.55 g cm^-3 | 5.5y | 6 | 2500 | 2.67555554 | 0.81017408 |
| M6POI3cut | Core | Core surface | Block | 1.55 g cm^-3 | 5.5y | 6 | 2500 | 2.45394929 | 0.74223072 |
| M6POI4 | Core | BS swab | Block | 1.55 g cm^-3 | 5.5y | 6 | 2500 | 2.84994082 | 0.9020064 |
| M6POI4cut | Core | BS cut | Block | 1.55 g cm^-3 | 5.5y | 6 | 2500 | 3.45349498 | 0.94848288 |
| M6POI5 | Core | BS swab | Block | 1.55 g cm^-3 | 5.5y | 6 | 2500 | 3.59922151 | 0.95985184 |
| M6POI6 | Core | BS swab | Block | 1.55 g cm^-3 | 5.5y | 6 | 2500 | 3.40215907 | 0.9481424 |
| M6POI6cut | Core | BS cut | Block | 1.55 g cm^-3 | 5.5y | 6 | 2500 | 3.35816452 | 0.93656192 |
| M6POI7 | Core | BS swab | Block | 1.55 g cm^-3 | 5.5y | 6 | 2500 | 2.31574333 | 0.81643552 |
| M6POI7cut | Core | BS cut | Block | 1.55 g cm^-3 | 5.5y | 6 | 2500 | 1.75342153 | 0.7345808 |
| M6POI8 | Core | BS swab | Block | 1.55 g cm^-3 | 5.5y | 6 | 2500 | 2.7663586 | 0.8876768 |
| M6POI9 | Core | Core shade | Block | 1.55 g cm^-3 | 5.5y | 6 | 2500 | 2.33362432 | 0.77941184 |
| M6S1 | Module | Module surface | Block | 1.55 g cm^-3 | 5.5y | 6 | 2500 | 3.64840173 | 0.95013952 |
| M6S2 | Module | Module surface | Block | 1.55 g cm^-3 | 5.5y | 6 | 2500 | 3.76310906 | 0.94451776 |
| M6S3 | Module | Module surface | Block | 1.55 g cm^-3 | 5.5y | 6 | 2500 | 3.99939651 | 0.9639376 |
| NAGRAPellets | Bentonite | Bulk interior | Pellets | 1.45 g cm^-3 | 0y | 0 | 2500 | 3.19779577 | 0.9070304 |
| NegativeControl2 | Control | Contamination control | Contamination control | Contamination control | 0y | 0 | 2500 | 2.37519742 | 0.85774944 |

## Alpha Diversity plots


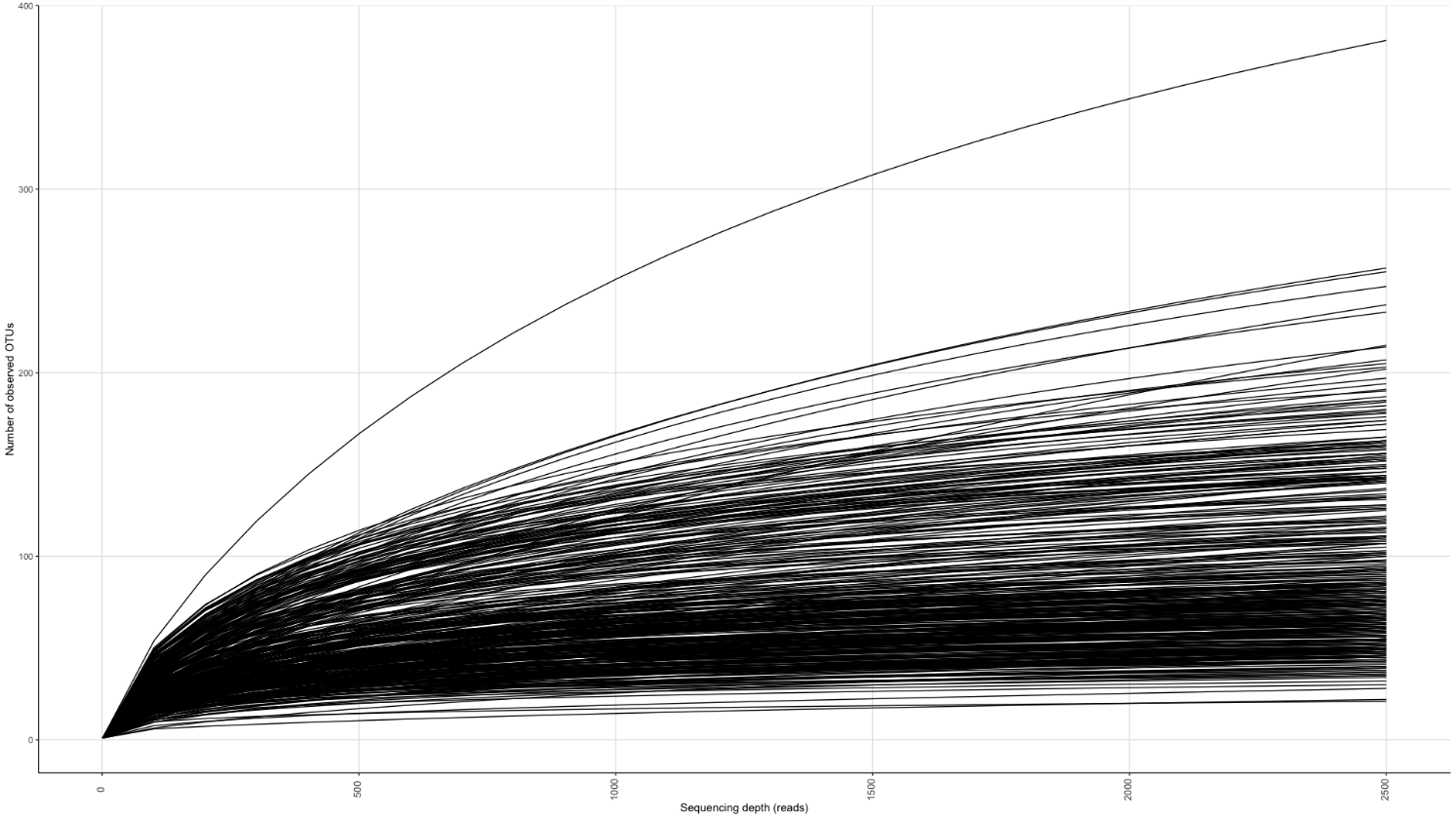


Figure S11 - Rarefaction curve of the analyzed 16S rRNA gene amplicon sequencing dataset. The number of reads was normalized to 2500 reads per sample.

1. Artificial Opalinus Clay porewater composition: 6.684075g/L NaCl, 3.408875 g/L Na_2_SO_4_, 1.9033 MgCl_2_ · 6 H_2_O, 1.787725 g/L CaCl_2_ · 2 H_2_O, 0.212125 g/L NaHCO_3_, 0.180075 KCl, 0.008925 KF [↑](#footnote-ref-1)
